# Matrix Stiffness Orchestrates Mesenchymal Stem Cell Lineage Commitment Toward Osteogenesis and Adipogenesis Through the PIEZO1/SP1/STC2 Axis

**DOI:** 10.1002/advs.77385

**Published:** 2026-09-06

**Authors:** Shuo Zhang, Siteng Li, Wenzhong Chen, Qingcheng Song, Haiyue Zhao, Peng Wang, Yiran Zhang, Wenquan Liang, Shaowei Zheng, Juan Wang, Wei Chen, Yanbin Zhu, Yingze Zhang

**Affiliations:** ^1^ School of Medicine Nankai University Tianjin China; ^2^ Department of Orthopedic Surgery The Third Hospital of Hebei Medical University Shijiazhuang Hebei China; ^3^ Department of Orthopaedic Trauma Centre for Orthopaedic Surgery The Third Affiliated Hospital Southern Medical University Guangzhou Guangdong China; ^4^ Department of Orthopedics Fuzhou University Affiliated Provincial Hospital Fuzhou Fujian China; ^5^ Department of Hand and Foot Surgery Shenzhen Second People's Hospital Shenzhen Guangdong China; ^6^ Department of Anatomy School of Basic Medical Sciences Southern Medical University Guangzhou Guangdong China; ^7^ Department of Sports Medicine and Rehabilitation Shenzhen Xinhua Hospital Peking University Shenzhen Hospital Shenzhen Guangdong China

**Keywords:** bone defect healing, matrix stiffness, mesenchymal stem cells, PIEZO1, STC2

## Abstract

Mesenchymal stem cells (MSCs) are critical for bone regeneration, and their osteogenic and adipogenic lineage balance is intricately regulated by cellular mechanotransduction. Through single‐cell RNA sequencing reanalysis of the public dataset GSE166824, primary mouse MSC functional assays, and in vivo genetic and bone defect models, this study identifies a novel PIEZO1/specificity protein 1 (SP1)/stanniocalcin 2 (STC2) signaling axis that drives matrix stiffness‐dependent MSC lineage commitment. Stiff matrices robustly direct MSCs toward osteogenic differentiation while inhibiting adipogenesis by activating the mechanosensitive ion channel PIEZO1. PIEZO1 activation triggers Ca^2^^+^ influx, leading to calcium/calmodulin‐dependent protein kinase II (CaMKII)‐dependent SP1 activation. SP1 shows enrichment at the promoter region of Stc2 and upregulates its expression. The secreted protein STC2 functions downstream of PIEZO1 on stiff matrices while retaining pro‐osteogenic and anti‐adipogenic activity when PIEZO1 remains inactive. In a mouse femoral bone defect model under hindlimb unloading, both stiff gelatin methacryloyl (GelMA) hydrogels and exogenous STC2 administration enhance early‐stage bone formation on day 7. Conversely, *Prrx1*‐lineage‐specific inducible conditional knockout (iCKO) of *Piezo1* abrogates the regulatory effects of matrix stiffness. These findings establish the PIEZO1/SP1/STC2 axis as a pivotal mechanosensitive signaling pathway for MSC fate determination, offering novel molecular targets for mechanically optimized bone regenerative biomaterials.

## Graphical abstract

Matrix stiffness directs mesenchymal stem cell lineage commitment through PIEZO1‐dependent activation of the SP1/STC2 pathway. Stiff matrices favor osteogenesis and suppress adipogenesis, whereas the loss of PIEZO1 function impairs early bone formation. STC2 retains lineage‐regulatory activity even when PIEZO1 is inactive. These findings identify a mechanosensitive signaling mechanism that may guide rational design of stiffness‐optimized biomaterials for bone regeneration.

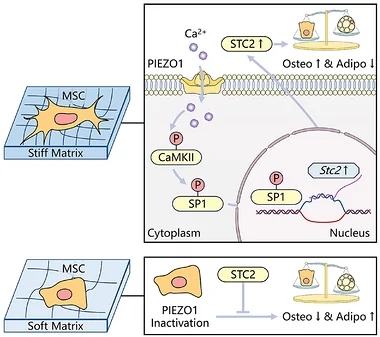

## 1 Introduction

Mechanical forces are essential physical stimuli that influence cellular behavior, thereby orchestrating organismal development and tissue homeostasis. The processes of mechanosensation and mechanotransduction are fundamental in converting mechanical signals into biochemical signals, enabling living organisms to perceive and respond to mechanical cues from both external and internal microenvironments [1]. Key biomechanical cues include extracellular matrix (ECM) stiffness, hydrostatic pressure, fluid shear stress (FSS), and tensile/compressive strain. Bone, a highly dynamic organ, is maintained through a continuous cycle of bone resorption and formation. Mechanical forces are extensively implicated in both the physiological and pathological regulation of bone, mediated by various cell‐surface mechanosensors such as PIEZO1 and transient receptor potential vanilloid 4 (TRPV4). The absence of mechanical stimulation severely compromises bone microstructure, increasing susceptibility to disuse osteoporosis and raising the risk of fractures [2]. Nonetheless, the specific effects and underlying molecular mechanisms by which biomechanical cues regulate bone homeostasis represent a critical area of ongoing investigation.

Bone‐resident mesenchymal stem cells (MSCs) exhibit self‐renewal capabilities and possess the potential for multilineage differentiation, playing a crucial role in bone injury repair [3]. Local microenvironments or niches communicate with MSCs through biomechanical signals to modulate cellular processes such as proliferation, migration, and differentiation [4]. In addition, MSCs residing in bone tissue secrete paracrine and autocrine factors that facilitate tissue regeneration and ameliorate the adverse microenvironment [5]. Importantly, the MSC secretome is intricately regulated by mechanical stimuli. Mechanical unloading upregulates the production of inflammatory cytokines by MSCs, which in turn promotes osteoclast differentiation and exacerbates bone resorption [6]. Stanniocalcin 2 (STC2), a secreted glycoprotein within the MSC secretome, has been implicated in directing MSC fate determination [5]. Despite the established role of STC2 in MSC biology, the upstream mechanosensory pathways and specific transcriptional regulators that mediate matrix stiffness‐dependent STC2 expression remain incompletely elucidated.

PIEZO1, a mechanosensitive cation channel, is critical for mechanical sensation and mechanotransduction across diverse cell types [7]. Within the musculoskeletal system, the influence of PIEZO1 activation on osteogenesis, chondrogenesis, and myogenesis has been extensively documented in previous research [8, 9]. In response to mechanical stimuli and PIEZO1 agonists, the opening of the PIEZO1 channel triggers Ca^2^^+^ influx, which subsequently modulates the function of transcription factors (TFs), regulates downstream signaling pathways, and ultimately influences the behavior and fate of MSCs [10]. Supporting its physiological importance, the conditional knockout (CKO) of PIEZO1 but not PIEZO2 in *Prrx1*‐lineage cells (a well‐characterized MSC population) resulted in a marked reduction in bone mass and the occurrence of spontaneous fractures in mice [11]. Beyond its established osteogenic mechanotransduction function, whether PIEZO1 controls MSC lineage commitment via uncharacterized secreted effectors and their corresponding transcriptional regulators requires further investigation.

ECM stiffness maintains a sustained spatiotemporal presence in the cellular microenvironment, forming the core pericellular mechanical niche and cell‐matrix coupling interface. A comprehensive understanding of cellular mechanisms for sensing matrix stiffness is therefore paramount for designing implantable biomaterials with optimal physicochemical properties for tissue engineering applications [12]. Harnessing appropriate mechanical signals to direct MSCs toward osteogenesis presents a promising strategy for enhanced bone regeneration. Extending the established paradigm of matrix stiffness‐driven, PIEZO1‐mediated osteogenesis, this study identifies a novel PIEZO1/specificity protein 1 (SP1)/STC2 signaling axis directing MSC lineage commitment. We therefore aim to elucidate how matrix stiffness regulates MSC osteogenic and adipogenic differentiation, and to delineate SP1‐mediated transcriptional control of *Stc2* as a downstream effector of PIEZO1 signaling governing MSC fate. Our findings advance the PIEZO1 mechanotransduction framework beyond canonical signaling pathways, by revealing a transcriptional and secretory regulatory axis that links mechanical cues to MSC fate determination.

## 2 Results

### 2.1 Single‐Cell RNA Sequencing (scRNA‐seq) Analysis of MSC Differentiation Potential

**FIGURE 1:**
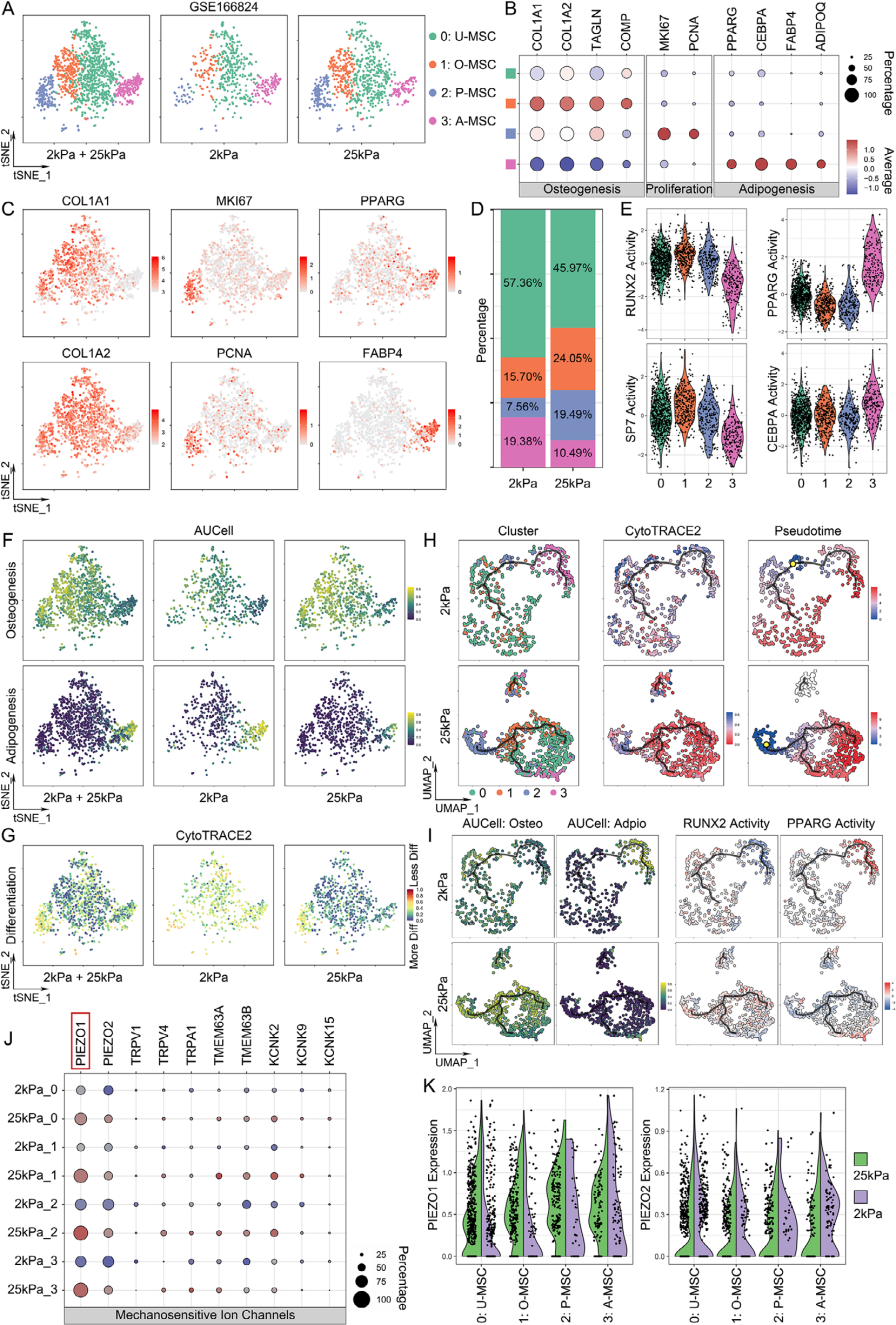
scRNA‐seq reanalysis uncovers the regulatory effects of matrix stiffness on MSC fate determination. (A) t‐SNE plots illustrating the subtypes of MSCs (GSE166824). (B) Dot plots depicting the specific marker genes in four cell clusters. (C) t‐SNE plots illustrating the expression patterns of marker genes (*COL1A1*, *COL1A2*, *MKI67*, *PCNA*, *PPARG*, *FABP4*) for the major cell clusters. (D) Bar graph quantifying the cell proportions across different matrix stiffness groups. (E) Violin plots characterizing the transcriptional activity of RUNX2, SP7, PPARG, and CEBPA in MSCs. (F) AUCell analysis evaluating the enrichment levels of osteogenic and adipogenic signature gene sets in MSCs. (G) CytoTRACE2 analysis assessing the stemness and differentiation potential of MSCs. (H, I) Monocle3 analysis reconstructing the developmental trajectories of MSCs, with the osteogenic/adipogenic scores and TF activity plotted along the pseudotime direction. (J) Dot plots exhibiting the expression of mechanosensitive ion channel genes in four cell clusters. (K) Violin plot depicting the expression of *PIEZO1* and *PIEZO2* in MSCs.

A publicly available scRNA‐seq dataset (GSE166824) was reanalyzed to investigate MSC heterogeneity on soft or stiff matrices. This dataset includes 1460 quality‐controlled single cells with no independent sample‐level replication. All findings from this analysis are hypothesis‐generating. t‐distributed stochastic neighbor embedding (t‐SNE) dimensionality reduction was applied to the filtered cell population. Unsupervised clustering delineated four transcriptionally distinct cell clusters (Clusters 0–3) (Figure 1A). Cells were annotated via the Seurat FindAllMarkers function and cross‐referenced with the CellMarker database, uncovering distinct functional specialization across the clusters: Cluster 0 (uncommitted MSCs, U‐MSCs); Cluster 1 (osteogenic MSCs, O‐MSCs) enriched for *COL1A1*, *COL1A2*, *TAGLN*, and *COMP*; Cluster 2 (proliferative MSCs, P‐MSCs) marked by *MKI67* and *PCNA*; Cluster 3 (adipogenic MSCs, A‐MSCs) characterized by *PPARG*, *CEBPA*, *FABP4*, and *ADIPOQ* (Figure 1B,C). Quantification of cluster proportions across different matrix stiffness conditions uncovered a stiffness‐associated pattern of lineage commitment. Soft matrices (2 kPa) were enriched for Cluster 3 (adipogenic MSCs), whereas stiff matrices (25 kPa) exhibited preferential enrichment of Cluster 1 (osteogenic MSCs) (Figure 1D).

Cluster‐specific TF activity was systematically quantified using the decoupleR package. Cluster 1 (osteogenic MSCs) displayed elevated activity of osteogenic regulatory transcription factors RUNX2 and SP7. In comparison, Cluster 3 (adipogenic MSCs) exhibited augmented activity of adipogenic lineage‐defining transcription factors PPARG and CEBPA (Figure 1E, Figure S1A). Notably, these lineage‐specific TFs showed restricted activity within their respective clusters, consistent with functional roles in stiffness‐directed MSC lineage commitment. To quantify the osteogenic and adipogenic differentiation potential of MSCs, AUCell signature scores were calculated using well‐characterized lineage‐specific marker gene sets. The osteogenic and adipogenic gene signature scores were enriched in Cluster 1 (osteogenic MSCs) and Cluster 3 (adipogenic MSCs), respectively. Compared with the soft matrix group, MSCs on stiff matrices exhibited higher osteogenic signature scores accompanied by reduced adipogenic signature scores, consistent with a stiffness‐associated shift in lineage commitment (Figure 1F).

Gene Ontology (GO) and Kyoto Encyclopedia of Genes and Genomes (KEGG) pathway enrichment analyses identified distinct functional signatures among the clusters. Specifically, terms related to ECM organization, cell cycle progression, and adipogenic lipid metabolism were selectively enriched in Cluster 1, Cluster 2, and Cluster 3, respectively (Figure S1B,C). These findings were further supported by Gene Set Enrichment Analysis (GSEA). In the comparison between Cluster 1 (osteogenic MSCs) and all other clusters, the terms “ossification” and “skeletal system morphogenesis” were positively enriched. Conversely, in the comparison between Cluster 3 (adipogenic MSCs) and all other clusters, the terms “lipid biosynthetic process” and “positive regulation of lipid metabolic process” were positively enriched (Figure S1D).

### 2.2 scRNA‐seq Analysis of MSC Lineage Progression Branches

The CytoTRACE2 package was utilized to evaluate the differentiation capacity of single cells, with higher CytoTRACE2 scores indicating stronger stemness. Cluster 2 (proliferative MSCs) exhibited the highest CytoTRACE2 scores among all clusters. Lineage‐committed clusters, including Cluster 1 (osteogenic MSCs) and Cluster 3 (adipogenic MSCs), displayed lower CytoTRACE2 scores. Notably, stiff matrices were correlated with reduced CytoTRACE2 scores, suggesting their contributory role in driving MSC lineage commitment (Figure 1G).

To map the differentiation trajectories of MSCs in response to varying matrix stiffness levels, trajectory analysis was performed using the Monocle3 package. The trajectory tree, rooted in Cluster 2 (proliferative MSCs), suggested two putative lineage branches corresponding to MSC lineage bifurcation (Figure 1H). The osteogenic lineage branch extended from the root cluster (Cluster 2) to the terminal cluster (Cluster 1), accompanied by a progressive upregulation of osteogenic signature scores and transcription factor RUNX2 activity along the differentiation trajectory. Conversely, the adipogenic lineage branch emanated from the same root cluster (Cluster 2) and terminated at Cluster 3, characterized by a progressive induction of adipogenic signature scores and transcription factor PPARG activity (Figure 1I). Collectively, these trajectory analyses indicated that matrix stiffness may direct proliferative MSCs toward osteogenic or adipogenic fates.

### 2.3 PIEZO1 as a Candidate Mechanosensor of Matrix Stiffness

The expression patterns of multiple mechanosensitive ion channels were systematically profiled across matrices of distinct stiffness and among different cellular clusters. The transcript levels of *PIEZO1*, rather than *PIEZO2*, were consistently upregulated on stiff matrices across all cell clusters (Figure 1J,K). This expression pattern identified PIEZO1 as a candidate matrix stiffness mechanosensor requiring further functional validation.

MSCs were isolated from the femurs of C57BL/6J mice. Following lineage‐specific induction in vitro, the isolated MSCs were successfully differentiated into osteoblasts, adipocytes, and chondrocytes, as evidenced by positive staining with Alizarin Red S (ARS), Oil Red O (ORO), and Alcian Blue (AB), respectively (Figure S2A–C). The cell surface antigen profile of the MSCs was evaluated by flow cytometry (FCM). More than 90% of the isolated MSCs were positive for the canonical MSC surface markers CD73, CD90, and CD105 (Figure S2D). In contrast, the frequencies of cells positive for non‐MSC markers, including CD31 (a marker for endothelial cells), CD34 (a marker for hematopoietic stem cells and endothelial cells), and CD45 (a marker for immune cells), were consistently below 5% in the isolated MSCs (Figure S2E). Collectively, these findings indicated that the isolated cells fulfilled the minimal characterization criteria for MSCs.

**FIGURE 2:**
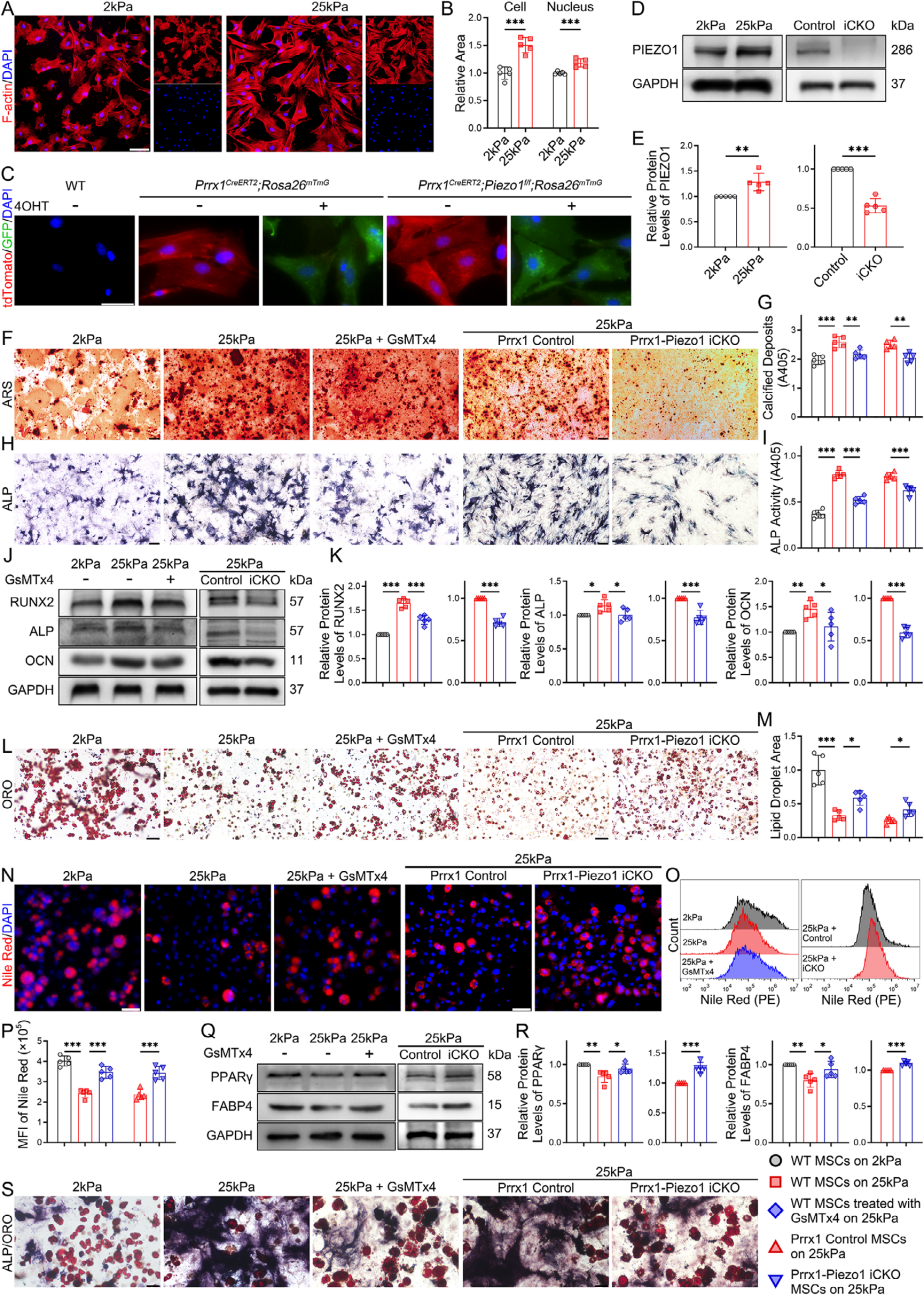
Stiff matrices promote osteogenesis and suppress adipogenesis in MSCs by activating PIEZO1. (A) F‐actin networks in MSCs visualized by phalloidin staining (scale bar, 100 µm). (B) Quantitative analysis of cell and nuclear area. (C) Fluorescence images of tdTomato and GFP expression in MSCs isolated from *Prrx1^CreERT2^;Rosa26^mTmG^* transgenic mice (scale bar, 50 µm). (D, E) Representative WB images and statistical analysis of PIEZO1 and GAPDH in MSCs. (F, G) ARS staining showing the calcified deposits of MSCs (scale bar, 200 µm). (H, I) ALP staining and ALP activity assays of MSCs (scale bar, 200 µm). (J, K) Representative WB images and statistical analysis of RUNX2, ALP, OCN, and GAPDH in MSCs. (L, M) ORO staining showing the lipid droplets of MSCs (scale bar, 100 µm). (N–P) Fluorescence images and FCM analysis of Nile Red staining of MSCs (scale bar, 50 µm). (Q, R) Representative WB images and statistical analysis of PPARγ, FABP4, and GAPDH in MSCs. (S) ALP staining and ORO staining evaluating the osteogenic and adipogenic differentiation potential of MSCs (scale bar, 50 µm). Cells were cultured on type I collagen‐coated polyacrylamide gels with elastic moduli of 2 kPa and 25 kPa. All experimental data are presented as mean ± SD. ^*^ *p* < 0.05, ^**^ *p* < 0.01, ^***^ *p* < 0.001. *n* = 5 biological replicates.

To investigate the impact of matrix stiffness on cytoskeletal organization, MSCs were cultured on hydrogel matrices with two distinct stiffness levels (2 and 25 kPa). MSCs cultured on stiff matrices displayed elongated F‐actin fibers and an extended morphology, whereas those on soft matrices showed a less spread, more rounded morphology (Figure 2A). Quantitatively, MSCs exhibited significantly larger average cell area and nuclear area on stiff matrices compared with those on soft matrices (Figure 2B). To further investigate the regulatory role of PIEZO1 in MSC lineage specification, MSCs isolated from *Prrx1*‐lineage‐specific *Piezo1* inducible CKO (iCKO) mice (*Prrx1^CreERT2^;Piezo1^f/f^* or *Prrx1^CreERT2^;Piezo1^f/f^;Rosa26^mTmG^*) and corresponding control mice (*Prrx1^CreERT2^* or *Prrx1^CreERT2^;Rosa26^mTmG^*) were cultured on stiff matrices and subjected to osteogenic and adipogenic induction protocols. Upon 4‐hydroxytamoxifen (4‐OHT) treatment, *Rosa26^mTmG^*‐positive cells exhibited a fluorescent transition from tdTomato to green fluorescent protein (GFP) expression, indicating the functional activity of the CreERT2 system (Figure 2C). Western blotting (WB) analysis validated that PIEZO1 protein levels were increased in MSCs cultured on stiff matrices compared with those on soft matrices, and were reduced in the iCKO group compared with the control group (Figure 2D,E).

### 2.4 Stiff Matrices Promote Osteogenesis and Inhibit Adipogenesis of MSCs by Activating PIEZO1

MSCs were cultured on matrices of different stiffness and subsequently induced toward osteogenic and adipogenic differentiation. ARS staining showed a greater abundance of calcified deposits on stiff matrices. Semi‐quantitative analysis of ARS staining indicated that the absorbance was higher in the stiff matrix group than in the soft matrix group (Figure 2F,G). Alkaline phosphatase (ALP) staining and ALP activity analysis further validated the pro‐osteogenic effects of stiff matrices (Figure 2H,I). WB assays revealed that stiff matrices upregulated the expression levels of core osteogenic markers runt‐related transcription factor 2 (RUNX2), ALP, and osteocalcin (OCN) in MSCs (Figure 2J,K). As expected, pharmacological blockade of mechanosensitive ion channels by GsMTx4 or genetic inactivation via *Piezo1* iCKO attenuated the osteogenic capacity of MSCs cultured on stiff matrices (Figure 2F–K).

Following adipogenic induction, lipid droplets accumulated extensively in MSCs, as visualized by ORO staining (Figure 2L,M) and Nile Red staining (Figure 2N–P). The protein levels of key adipogenic regulators, peroxisome proliferator‐activated receptor gamma (PPARγ) and fatty acid‐binding protein 4 (FABP4), were assessed by WB assays (Figure 2Q,R). The adipogenic potential of MSCs was significantly attenuated in the stiff matrix group compared with the soft matrix group. Conversely, GsMTx4 treatment or *Piezo1* iCKO potently promoted adipogenic differentiation of MSCs (Figure 2L–R).

Culture in bipotential differentiation medium allowed concurrent osteogenic and adipogenic differentiation. Compared with soft matrices, stiff matrices strongly favored osteogenesis while simultaneously suppressing adipogenic differentiation in MSCs (Figure 2S).

### 2.5 PIEZO1 Activation Promotes Osteogenesis and Inhibits Adipogenesis of MSCs

To further elucidate the regulatory effects of PIEZO1 on MSC differentiation in vitro, primary mouse MSCs were treated with the PIEZO1‐specific agonist Yoda1 and its competitive antagonist Dooku1. Yoda1 treatment facilitated the accumulation of calcified deposits and augmented ALP activity in MSCs undergoing osteogenic differentiation, as demonstrated by ARS staining (Figure S3A,B), ALP staining (Figure S3C), and ALP activity assays (Figure S3D). WB analysis indicated that Yoda1 elevated the expression levels of RUNX2, ALP, and OCN (Figure S3E,F). Conversely, Dooku1 partially mitigated the pro‐osteogenic effects induced by Yoda1 (Figure S3A–F).

Moreover, MSCs were stimulated with Yoda1 in the presence and absence of Dooku1 to determine the influence of PIEZO1 on the adipogenic potential of MSCs in vitro. ORO staining (Figure S3G,H) and Nile Red staining (Figure S3I–K) revealed that Yoda1 reduced the number and size of lipid droplets in MSCs undergoing adipogenic differentiation. WB analysis demonstrated that Yoda1 treatment resulted in the downregulation of PPARγ and FABP4 (Figure S3L,M). Dooku1 also partially counteracted the inhibitory effects of Yoda1 on MSC adipogenesis (Figure S3G–M).

Under bipotential induction conditions, activation of PIEZO1 by Yoda1 concurrently facilitated osteogenesis and suppressed adipogenesis of MSCs (Figure S3N).

### 2.6 Stiff Hydrogel Promotes Early‐Stage Bone Defect Healing in Mice under Mechanical Unloading Conditions

Gelatin methacryloyl (GelMA) hydrogels with varying stiffness were fabricated by modulating the GelMA concentration. Stress–strain analyses revealed that the compressive modulus increased in a concentration‐dependent manner. The compressive moduli of GelMA hydrogels at concentrations of 5%, 7.5%, 10%, 12.5%, 15%, and 17.5% were determined to be 3.58 ± 0.55, 8.41 ± 1.14, 13.42 ± 1.20, 17.72 ± 0.86, 24.25 ± 2.60, and 36.62 ± 3.94 kPa, respectively (Figure S4A,B).

**FIGURE 3:**
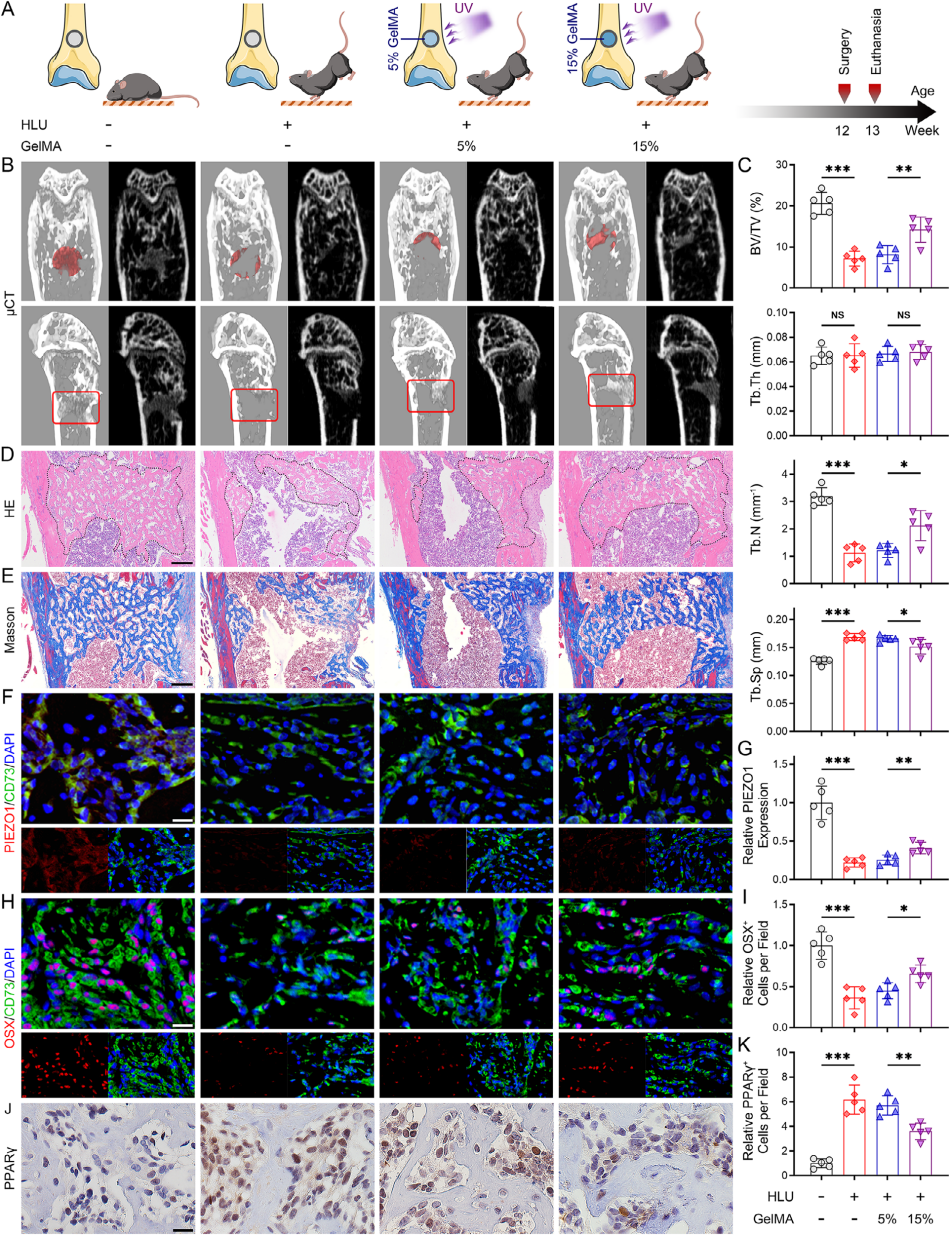
Stiff hydrogel promotes early‐stage bone defect healing in mice under mechanical unloading conditions. (A) Experimental scheme of the mouse femoral bone defect model. (B, C) µCT images of newly regenerated bone tissue in the femoral defect region. (D, E) HE staining and Masson's trichrome staining (scale bar, 200 µm). (F, G) IF staining for CD73 (green) and PIEZO1 (red) (scale bar, 20 µm). (H, I) IF staining for CD73 (green) and OSX (red) (scale bar, 20 µm). (J, K) IHC staining for PPARγ in femoral bone defects (scale bar, 20 µm). All experimental data are presented as mean ± SD. ^*^ *p* < 0.05, ^**^ *p* < 0.01, ^***^ *p* < 0.001, NS: not significant. *n* = 5 mice per group.

To examine the in vivo effects of matrix stiffness on bone regeneration under mechanical unloading conditions, a murine femoral bone defect model was established in conjunction with hindlimb unloading (HLU) (Figure 3A). As visualized by micro‐computed tomography (µCT) analysis along with hematoxylin and eosin (HE) staining, the HLU group exhibited a markedly reduced area of newly formed bone tissue compared with the ground control group (Figure 3B,D). Quantitative assessments of µCT‐derived bone parameters, including bone volume/total volume (BV/TV), trabecular number (Tb.N), and trabecular separation (Tb.Sp), consistently validated these observations (Figure 3C). Masson's trichrome staining showed reduced collagen deposition within the defect area in the HLU group, suggesting that mechanical unloading adversely affected new bone formation (Figure 3E). Under mechanical unloading conditions, the implantation of the stiff hydrogel (15% GelMA) resulted in more robust trabecular bone formation at the defect site compared with the implantation of the soft hydrogel (5% GelMA) (Figure 3B–E).

Immunostaining was conducted to assess the expression levels of PIEZO1, Osterix/SP7 (OSX), and PPARγ within the bone defect region. The immunofluorescence (IF) signals for PIEZO1 were markedly diminished in CD73‐positive cells from the HLU group, whereas these signals were robustly enhanced in the stiff hydrogel group compared with the soft hydrogel group (Figure 3F,G). Mechanical unloading resulted in a decrease in the number of OSX‐positive cells and an increase in PPARγ‐positive cells at the bone defect site (Figure 3H–K). In comparison to the soft hydrogel group, the stiff hydrogel group exhibited a significantly increased number of OSX‐positive cells, accompanied by a decreased number of PPARγ‐positive cells (Figure 3H–K). Collectively, these in vivo findings demonstrated that the stiff hydrogel promoted early‐stage bone defect healing by increasing PIEZO1 expression, thereby facilitating osteogenic differentiation while suppressing adipogenic differentiation of endogenous MSCs under mechanical unloading conditions.

### 2.7 *Prrx1*‐Lineage‐Specific *Piezo1* iCKO Suppresses Early‐Stage Bone Defect Healing in Mice and Counteracts the Pro‐Regenerative Effects of Stiff Hydrogel

**FIGURE 4:**
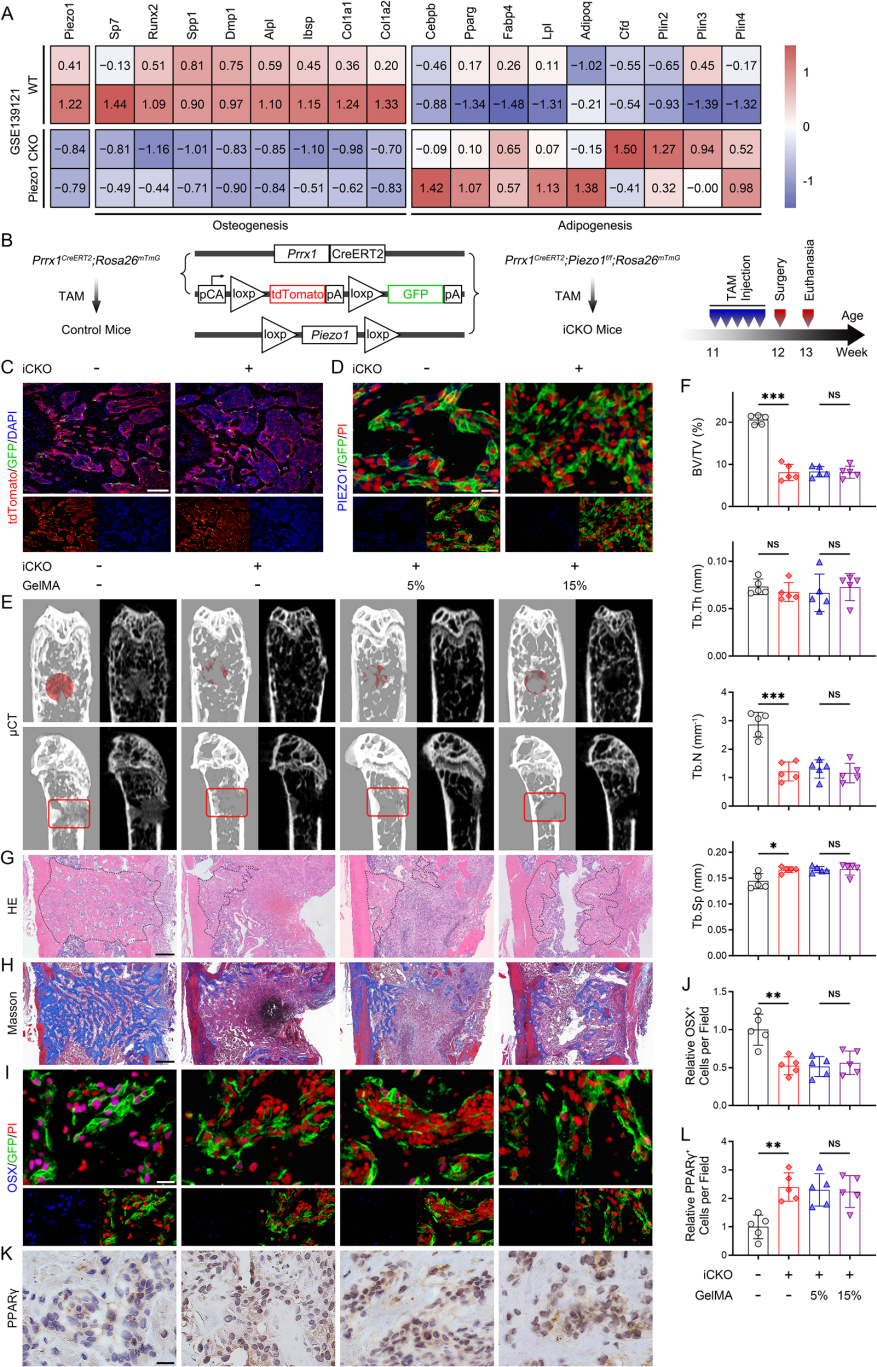
*Prrx1*‐lineage‐specific *Piezo1* iCKO suppresses early‐stage bone defect healing in mice and counteracts the pro‐regenerative effects of stiff hydrogel. (A) Heatmap revealing the relative expression levels of osteogenic and adipogenic marker genes in mouse bone tissues (GSE139121). (B) Experimental scheme of the femoral bone defect model in *Piezo1* iCKO mice. (C) IF staining for GFP (green) and tdTomato (red) of femurs (scale bar, 200 µm). (D) IF staining for GFP (green) and PIEZO1 (blue) of femoral bone defects (scale bar, 20 µm). (E, F) µCT images of newly regenerated bone tissue in the femoral defect region. (G, H) HE staining and Masson's trichrome staining (scale bar, 200 µm). (I, J) IF staining for GFP (green) and OSX (blue) (scale bar, 20 µm). (K, L) IHC staining for PPARγ in femoral bone defects (scale bar, 20 µm). All experimental data are presented as mean ± SD. ^*^ *p* < 0.05, ^**^ *p* < 0.01, ^***^ *p* < 0.001, NS: not significant. *n* = 5 mice per group.

A previously published dataset (GSE139121) was analyzed to evaluate the gene expression profiles in bone tissue from wild‐type (WT) and *Prrx1*‐lineage‐specific *Piezo1* CKO mice. Compared with WT controls, *Piezo1* CKO mice showed reduced expression of *Piezo1* and osteogenic signature genes (e.g., *Sp7*, *Runx2*, *Spp1*, *Dmp1*, *Alpl*, *Ibsp*, *Col1a1*), alongside increased expression of adipogenic signature genes (e.g., *Cebpb*, *Pparg*, *Fabp4*, *Lpl*, *Adipoq*, *Cfd*) (Figure 4A). To further substantiate the functional contribution of PIEZO1 in bone defect healing, we generated a *Prrx1*‐lineage‐specific *Piezo1* iCKO mouse line and conducted femoral bone defect surgery (Figure 4B). After tamoxifen (TAM) treatment, GFP was specifically expressed in *Prrx1*‐lineage cells, with the majority of GFP‐positive cells residing on the surfaces of trabecular bone (Figure 4C). IF staining revealed that PIEZO1 signals were almost undetectable in GFP‐positive cells of *Piezo1* iCKO mice (Figure 4D). µCT analysis, along with HE staining and Masson's trichrome staining, consistently demonstrated that *Piezo1* iCKO mice exhibited a reduced area of newly formed bone tissue at the defect site, compared with corresponding control mice (Figure 4E–H). Notably, the implantation of either the stiff or soft hydrogel failed to enhance bone formation in *Piezo1* iCKO mice (Figure 4E–H).

Accordingly, there was a reduction in the number of OSX‐positive osteoblast‐like cells in *Piezo1* iCKO mice (Figure 4I,J). In contrast, PPARγ‐positive cells accumulated significantly at the defect site of *Piezo1* iCKO mice (Figure 4K,L). However, no significant differences were observed between the stiff and soft hydrogel treatment groups (Figure 4I–L). Taken together, these results provided compelling evidence that PIEZO1 is essential for bone defect healing, and the modulatory effect of matrix stiffness on bone regeneration is nullified in the absence of PIEZO1.

### 2.8 STC2 Acts as a Candidate Mechanosensitive Effector Regulated by PIEZO1 Activation in MSCs

**FIGURE 5:**
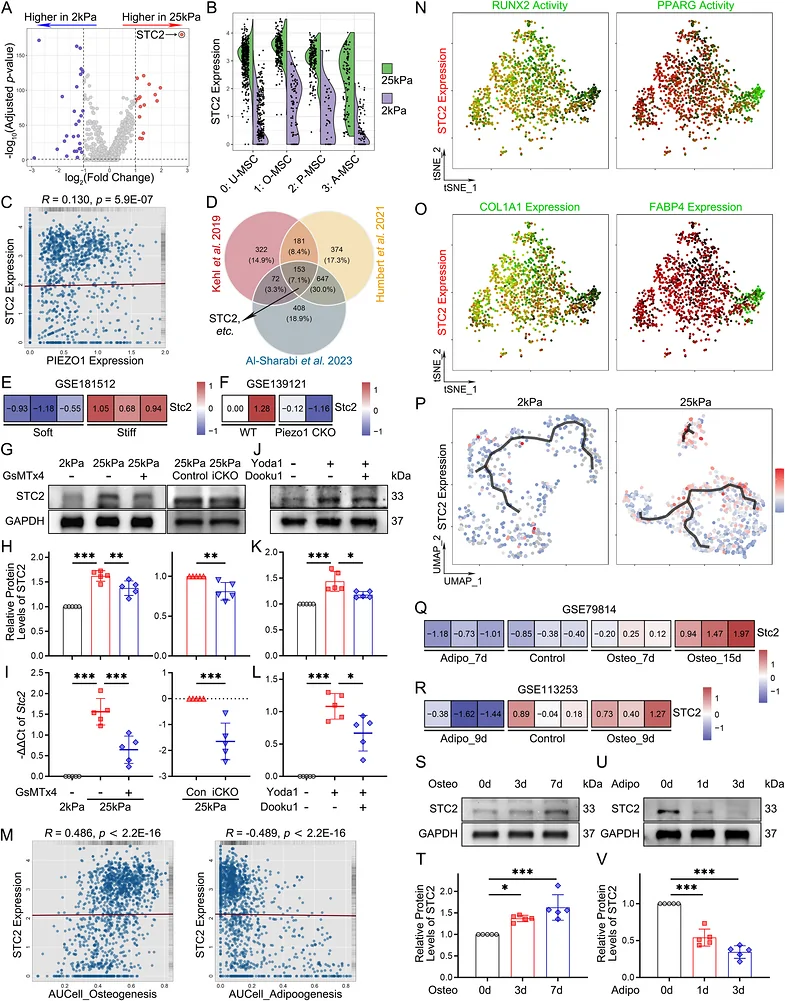
scRNA‐seq reveals STC2 as a candidate mechanosensitive effector regulated by PIEZO1 activation. (A) Volcano plot displaying DEGs in MSCs cultured on stiff versus soft matrices. (B) Violin plots depicting the expression of *STC2* in MSCs. (C) Cell‐level exploratory correlation analysis of *STC2* expression and *PIEZO1* expression. (D) Venn diagram identifying STC2 as a component of the MSC secretome. (E) Heatmap showing the relative expression levels of *Stc2* in rat MSCs (GSE181512). (F) Heatmap showing the relative expression levels of *Stc2* in mouse bone tissues (GSE139121). (G, H) Representative WB images and statistical analysis of STC2 and GAPDH in MSCs. (I) The mRNA levels of *Stc2* in MSCs. (J, K) Representative WB images and statistical analysis of STC2 and GAPDH in MSCs. (L) The mRNA levels of *Stc2* in MSCs. (M) Cell‐level exploratory correlation analysis of *STC2* expression and AUCell osteogenic or adipogenic scores. (N) t‐SNE plots illustrating *STC2* expression (red gradient) overlaid with the activity levels of RUNX2 or PPARG (green gradient). (O) t‐SNE plots illustrating *STC2* expression (red gradient) overlaid with the expression levels of *COL1A1* or *FABP4* (green gradient). (P) MSC developmental trajectories with *STC2* expression plotted along the pseudotime direction. (Q) Heatmap showing the expression levels of *Stc2* in mouse MSCs (GSE79814). (R) Heatmap showing the expression levels of *STC2* in human MSCs (GSE113253). (S‐V) Representative WB images and statistical analysis of STC2 and GAPDH in MSCs. All experimental data are presented as mean ± SD. ^*^ *p* < 0.05, ^**^ *p* < 0.01, ^***^ *p* < 0.001. *n* = 5 biological replicates for experiments on primary MSCs.

To explore candidate mechanosensitive pathways downstream of PIEZO1, scRNA‐seq data from MSCs cultured on matrices with different stiffness (GSE166824) were subjected to further analysis. The volcano plot identified STC2 as one of the markedly upregulated genes in MSCs cultured on stiff matrices (Figure 5A). The violin plot showed elevated expression of STC2 in MSCs under stiff matrix conditions (Figure 5B). Furthermore, single‐cell correlation analysis detected a positive correlation between the expression of STC2 and PIEZO1 within this dataset (Figure 5C). Secretome profiling across three independent, previously published datasets consistently identified STC2 as a secreted factor from human bone marrow MSCs (Figure 5D) [13, 14, 15].

Analysis of publicly available RNA‐seq datasets (GSE181512 and GSE139121) showed that STC2 expression was elevated in MSCs cultured on stiff matrices compared with those on soft matrices (Figure 5E), as well as in WT bone tissue compared with *Prrx1‐lineage‐specific Piezo1* CKO bone tissue (Figure 5F). Consistently, GsMTx4 treatment or *Piezo1* iCKO abrogated the stiffness‐induced upregulation of STC2 protein (Figure 5G,H), and significantly reduced its mRNA levels (Figure 5I). In contrast, the pharmacological activation of PIEZO1 using Yoda1 enhanced both the protein and mRNA levels of STC2, an effect that was partially mitigated by Dooku1 (Figure 5J–L), further supporting that STC2 expression is regulated by PIEZO1 activation.

Single‐cell correlation analysis further showed that STC2 expression was positively associated with AUCell osteogenic scores and negatively associated with AUCell adipogenic scores within this dataset (Figure 5M). As shown in the feature plots, STC2 expression was higher in MSCs with high RUNX2 activity and *COL1A1* expression, but lower in those with high PPARG activity and *FABP4* expression (Figure 5N,O). Pseudotime trajectory analysis of MSCs showed a progressive increase in STC2 expression along the osteogenic developmental trajectory, which was more prominent under stiff matrix conditions (Figure 5P). Reanalysis of gene expression datasets (GSE79814 and GSE113253) showed that STC2 expression was increased during osteogenic differentiation and decreased during adipogenic differentiation (Figure 5Q,R). In vitro differentiation assays of MSCs confirmed that STC2 protein levels markedly increased during osteogenic differentiation (Figure 5S,T) and decreased during adipogenic differentiation (Figure 5U,V).

Collectively, these results identified STC2 as a stiffness‐sensitive secreted factor that is regulated by PIEZO1 activation and functionally involved in the osteogenic and adipogenic lineage commitment of MSCs.

### 2.9 STC2 Promotes Osteogenesis and Suppresses Adipogenesis of MSCs

To evaluate the effect of STC2 on MSC viability, Cell Counting Kit‐8 (CCK‐8) assays were performed on MSCs treated with increasing concentrations of STC2 (0, 20, 50, 100, 200, 500, 1000 ng/mL) for 24, 48, and 72 h. As presented in Figure S5A–C, STC2 increased CCK‐8 metabolic activity across several tested concentrations, with the strongest effect observed around 500 ng/mL.

**FIGURE 6:**
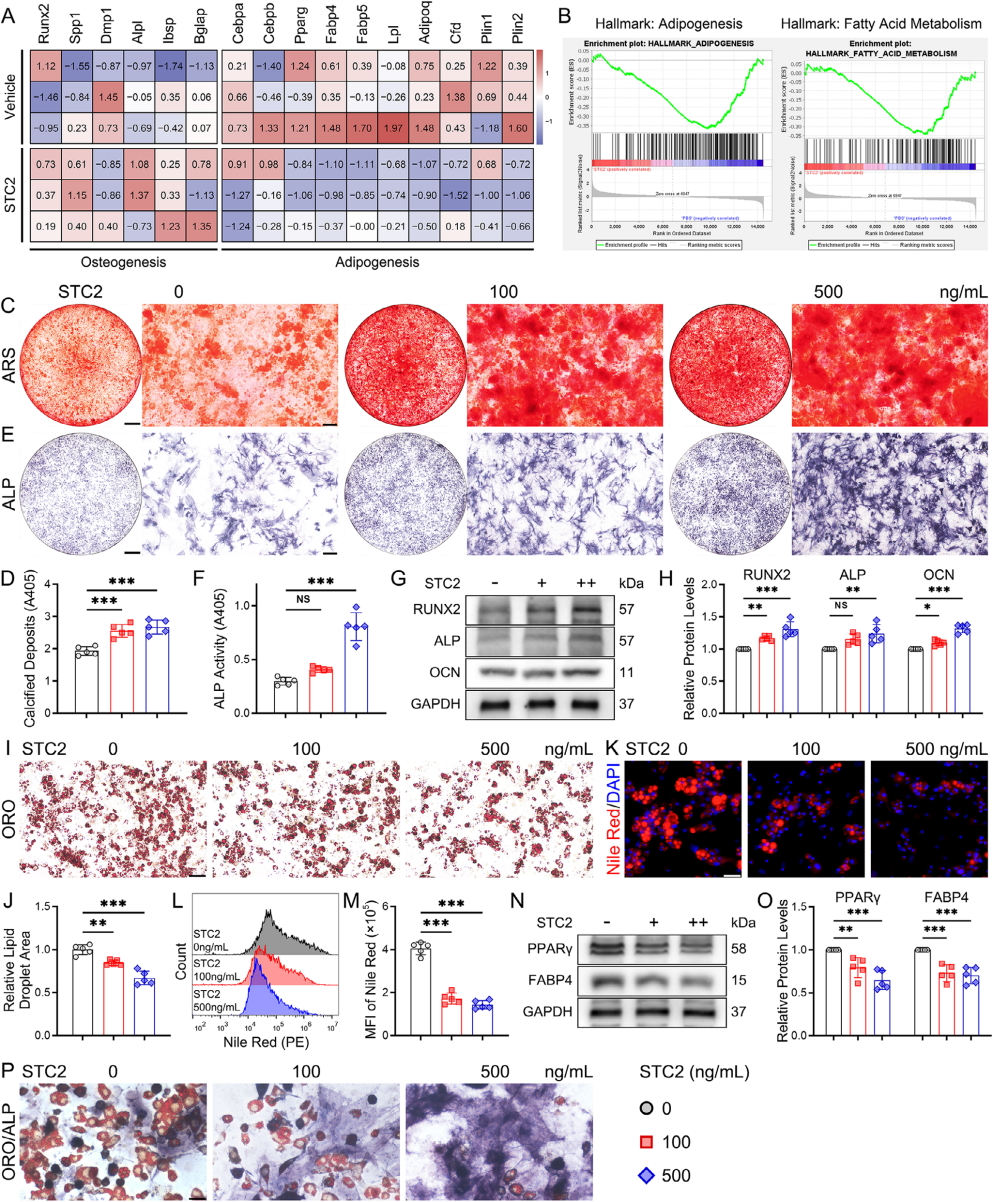
STC2 promotes osteogenesis and suppresses adipogenesis in MSCs. (A) Heatmap revealing the relative expression levels of osteogenic and adipogenic marker genes in MSCs. (B) GSEA plots depicting the enrichment of gene sets related to adipogenesis from RNA‐seq data. (C, D) ARS staining showing the calcified deposits of MSCs (left scale bar, 2 mm; right scale bar, 200 µm). (E, F) ALP staining and ALP activity assays of MSCs (left scale bar, 2 mm; right scale bar, 200 µm). (G, H) Representative WB images and statistical analysis of RUNX2, ALP, OCN, and GAPDH in MSCs. (I, J) ORO staining showing the lipid droplets of MSCs (scale bar, 100 µm). (K–M) Fluorescence images and FCM analysis of Nile Red staining of MSCs (scale bar, 50 µm). (N, O) Representative WB images and statistical analysis of PPARγ, FABP4, and GAPDH in MSCs. (P) ALP staining and ORO staining evaluating the osteogenic and adipogenic differentiation potential of MSCs (scale bar, 50 µm). Cells were cultured on tissue culture polystyrene (TCPS) plates. All experimental data are presented as mean ± SD. ^*^ *p* < 0.05, ^**^ *p* < 0.01, ^***^ *p* < 0.001, NS: not significant. *n* = 5 biological replicates.

RNA‐seq was performed on MSCs treated with either STC2 or vehicle under bipotential differentiation conditions to delineate the molecular mechanisms underlying STC2‐mediated MSC lineage commitment. Heatmap analysis of lineage‐specific marker genes revealed a distinct transcriptional profile: STC2 treatment resulted in the upregulation of osteogenic genes (e.g., *Runx2*, *Spp1*, *Alpl*, *Ibsp*, *Bglap*) and the downregulation of adipogenic genes (e.g., *Pparg*, *Fabp4*, *Lpl*, *Adipoq*, *Cfd*) compared with vehicle‐treated controls (Figure 6A). Consistent with these gene expression changes, GSEA demonstrated that the “adipogenesis” and “fatty acid metabolism” gene sets were negatively enriched in STC2‐treated MSCs relative to controls (Figure 6B).

To further elucidate the regulatory role of STC2 in MSC lineage commitment in vitro, MSCs were treated with recombinant STC2 protein. Exogenous STC2 elicited a robust osteogenic response compared with vehicle‐treated controls, as demonstrated by ARS staining (Figure 6C,D), ALP staining (Figure 6E), and ALP activity assays (Figure 6F). WB analysis confirmed that STC2 treatment markedly upregulated the expression levels of RUNX2, ALP, and OCN in a dose‐dependent manner (Figure 6G,H).

Moreover, MSCs were treated with STC2 to assess its impact on adipogenic differentiation potential. As visualized by ORO staining (Figure 6I,J) and Nile Red staining (Figure 6K–M), treatment with STC2 significantly reduced the number and size of lipid droplets in MSCs induced toward adipogenic differentiation. These phenotypic alterations were corroborated by WB analysis, which demonstrated a marked downregulation of PPARγ and FABP4 expression in STC2‐treated MSCs (Figure 6N,O).

In bipotential differentiation assays, STC2 supplementation notably shifted the differentiation balance of MSCs toward osteogenesis while diminishing adipogenesis, thereby confirming the dual regulatory role of STC2 in directing MSC lineage specification (Figure 6P).

### 2.10 STC2 Promotes Osteogenesis and Suppresses Adipogenesis of MSCs Under PIEZO1‐inactivated Conditions

To determine whether STC2 exerts its lineage‐regulatory effects independently of PIEZO1 activation, the differentiation potential of MSCs was assessed under two distinct conditions of PIEZO1 inactivation: culture on soft matrices or culture on stiff matrices combined with GsMTx4 treatment. Under both PIEZO1‐inactivated conditions, STC2 supplementation robustly enhanced the osteogenic differentiation capacity of MSCs, as evidenced by ARS staining (Figure S6A,B), ALP staining, and ALP activity assays (Figure S6C,D). Conversely, STC2 treatment substantially suppressed adipogenic differentiation in MSCs with inactivated PIEZO1. Both ORO staining (Figure S6E,F) and Nile Red staining (Figure S6G,H) revealed a prominent decrease in the number and size of lipid droplets in STC2‐treated MSCs. In parallel bipotential differentiation assays, the addition of STC2 further shifted the differentiation balance toward osteogenesis while repressing adipogenesis (Figure S6I). These findings collectively demonstrated that STC2 promotes osteogenesis and suppresses adipogenesis in MSCs under PIEZO1‐inactivated conditions, implying that its lineage‐regulatory function is at least partially independent of PIEZO1‐mediated mechanotransduction.

### 2.11 STC2 Promotes Early‐Stage Bone Defect Healing in Mice under Mechanical Unloading Conditions

**FIGURE 7:**
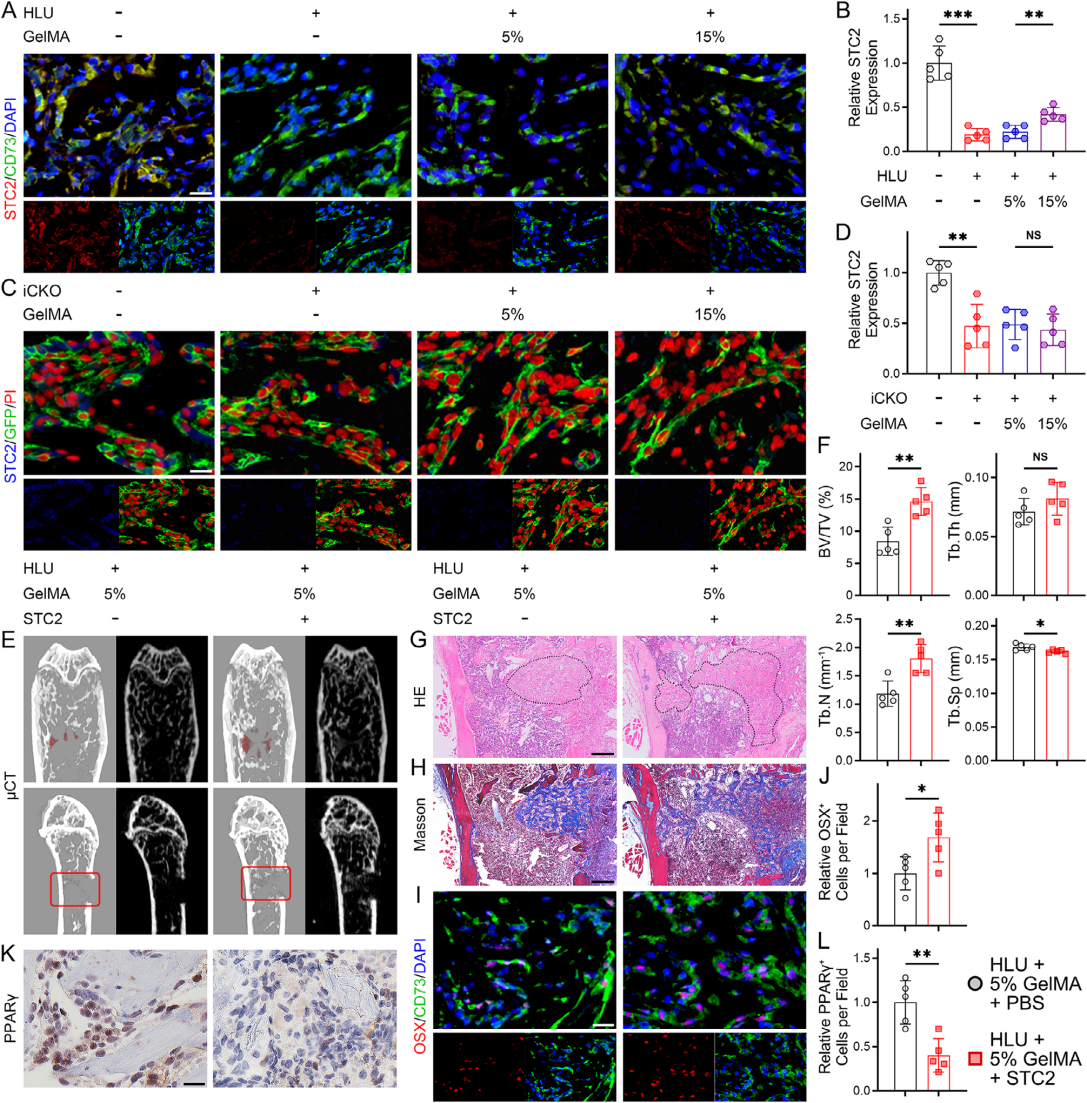
STC2 promotes early‐stage bone defect healing in mice under mechanical unloading conditions. (A, B) IF staining for CD73 (green) and STC2 (red) of femoral bone defects (scale bar, 20 µm). (C, D) IF staining for GFP (green) and STC2 (blue) of femoral bone defects (scale bar, 20 µm). (E, F) µCT images of newly regenerated bone tissue in the femoral defect region. (G, H) HE staining and Masson's trichrome staining (scale bar, 200 µm); (I, J) IF staining for CD73 (green) and OSX (red) (scale bar, 20 µm); (K, L) IHC staining for PPARγ in femoral bone defects (scale bar, 20 µm). All experimental data are presented as mean ± SD. ^*^ *p* < 0.05, ^**^ *p* < 0.01, ^***^ *p* < 0.001, NS: not significant. *n* = 5 mice per group.

IF staining was performed on femoral defect sections to assess STC2 expression. The HLU group exhibited markedly reduced expression of STC2 in CD73‐positive cells compared with the ground control group (Figure 7A,B). Under conditions of HLU‐induced mechanical unloading, the implantation of the stiff hydrogel upregulated STC2 expression compared with the soft hydrogel treatment (Figure 7A,B). Compared with corresponding control mice, *Prrx1*‐lineage‐specific *Piezo1* iCKO mice displayed markedly reduced expression of STC2 in GFP‐positive cells within the defect site (Figure 7C,D). Notably, the implantation of the stiff hydrogel did not result in enhanced STC2 expression in *Piezo1* iCKO mice, with no apparent differences between the stiff and soft hydrogel groups (Figure 7C,D).

To investigate the in vivo pro‐osteogenic effects of STC2 on bone healing, 5% GelMA hydrogel loaded with either STC2 or vehicle was implanted into the bone defect. Quantitative analysis of key trabecular bone parameters derived from µCT scanning, including BV/TV, Tb.N, and Tb.Sp, consistently supported the pro‐osteogenic effects of STC2‐loaded hydrogel (Figure 7E,F). Histological evaluations using HE and Masson's trichrome staining demonstrated that the STC2‐treated group exhibited markedly enhanced collagen deposition and de novo bone formation within the defect region compared with the vehicle control group (Figure 7G,H). Within the bone defect area, the STC2‐loaded hydrogel group showed a substantial increase in the number of OSX‐positive osteoblast‐like cells (Figure 7I,J), coupled with a significant reduction in the number of PPARγ‐positive cells (Figure 7K,L).

### 2.12 Transcription Factor SP1 Upregulates STC2 Expression by Regulating Promoter Activity

**FIGURE 8:**
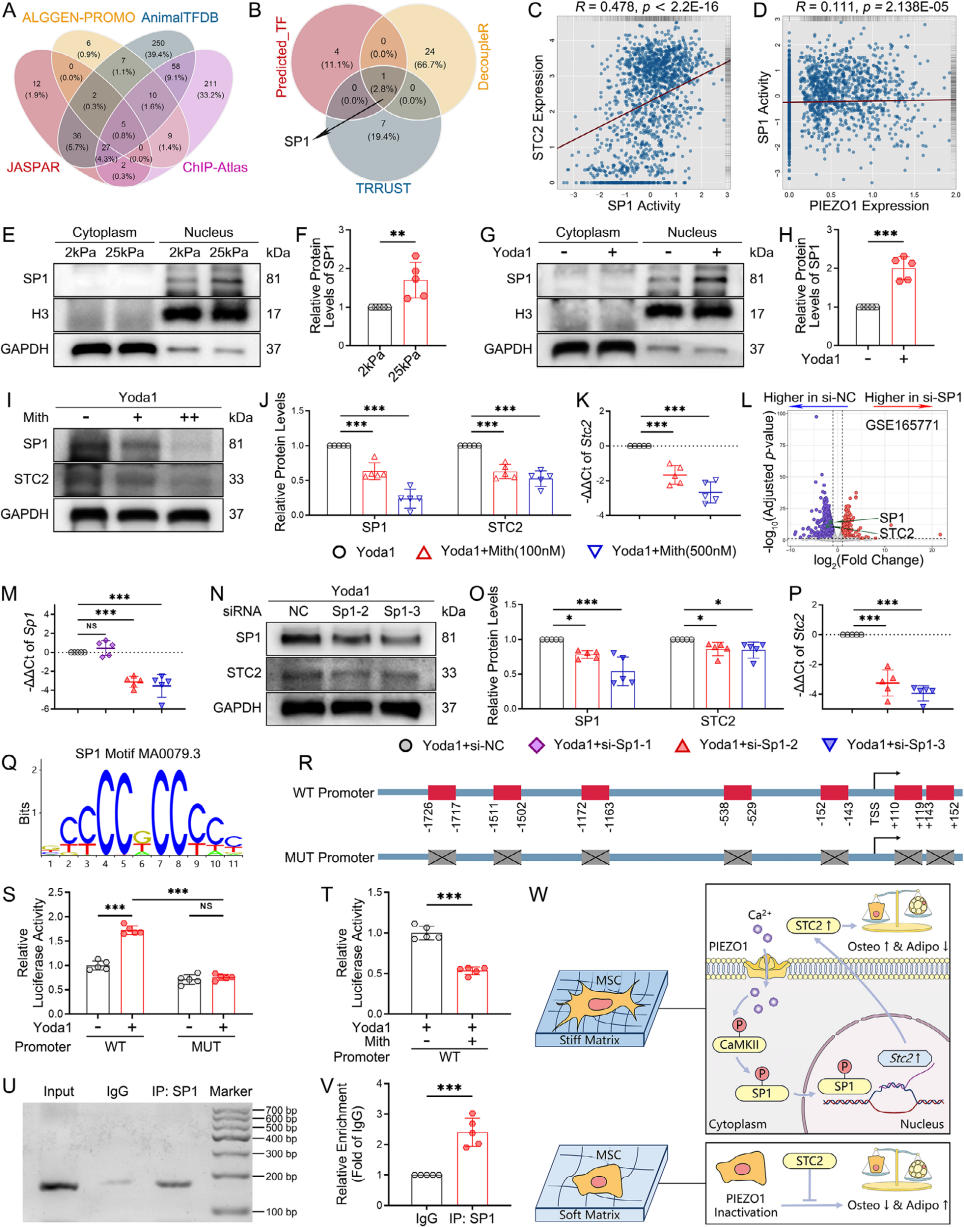
Transcription factor SP1 upregulates STC2 expression by regulating promoter activity. (A) Venn diagram identifying the putative TFs predicted to regulate *Stc2* expression using the JASPAR, ALGGEN‐PROMO, AnimalTFDB, and ChIP‐Atlas databases. (B) Venn diagram further refining the prediction of the putative TFs for *Stc2* regulation using the decoupleR package and TRRUST database. (C) Cell‐level exploratory correlation analysis of *STC2* expression and SP1 activity. (D) Cell‐level exploratory correlation analysis of SP1 activity and *PIEZO1* expression. (E–H) Representative WB images and statistical analysis of SP1, Histone H3, and GAPDH in MSCs. (I, J) Representative WB images and statistical analysis of SP1, STC2, and GAPDH in MSCs. (K) The mRNA levels of *Stc2* in MSCs. (L) Volcano plot displaying DEGs in HEK293 cells transfected with *SP1* siRNA versus NC siRNA (GSE165771). (M) The mRNA levels of *Sp1* in MSCs. (N, O) Representative WB images and statistical analysis of SP1, STC2, and GAPDH in MSCs. (P) The mRNA levels of *Stc2* in MSCs. (Q) The consensus DNA‐binding motif of transcription factor SP1. (R) Schematic diagrams of WT and MUT *Stc2* promoter reporter constructs. Red boxes represent predicted SP1 binding sites relative to the transcription start site (TSS); gray boxes represent deletion mutations of the potential SP1 binding sites. (S, T) Luciferase reporter assays demonstrating the functional regulation of the *Stc2* promoter by SP1. (U, V) Detection of SP1 enrichment at the *Stc2* promoter region by ChIP‐PCR, followed by electrophoresis on a GelRed‐containing agarose gel. (W) Schematic illustration showing the contribution of matrix stiffness‐mediated PIEZO1 activation to MSC fate determination by promoting osteogenesis and inhibiting adipogenesis via the Ca^2^^+^/CaMKII/SP1/STC2 signaling axis. All experimental data are presented as mean ± SD. ^*^ *p* < 0.05, ^**^ *p* < 0.01, ^***^ *p* < 0.001, NS: not significant. *n* = 5 biological replicates for experiments on primary MSCs. *n* = 5 independent experiments on HEK293T cells.

TFs with potential binding affinity to the mouse *Stc2* promoter were predicted using the JASPAR, ALGGEN‐PROMO, AnimalTFDB, and ChIP‐Atlas databases. Through intersection analysis, five candidate TFs were identified: SP1, MYOD1, MYOG, PAX5, and POU2F1 (Figure 8A). Differential TF activity analysis, conducted via decoupleR, identified 25 candidate TFs, among which 17 were upregulated and 8 were downregulated (Figure S7A). Differentially expressed genes (DEGs) were further analyzed for TF target enrichment using the TRRUST database. By integrating these predictive results, SP1 emerged as a key candidate TF mediating stiffness‐dependent regulation of *STC2* expression (Figure 8B). The activities of SP1, MYOD1, MYOG, PAX5, and POU2F1 were positively correlated with *STC2* expression, with SP1 activity exhibiting the strongest correlation (Figure 8C, Figure S7B). Moreover, SP1 activity was positively correlated with *PIEZO1* expression (Figure 8D). Notably, SP1 transcriptional activity, rather than its overall expression level, was significantly enhanced under conditions of stiff matrix culture (Figure S7C,D).

WB analysis, following the fractionation of nuclear and cytoplasmic proteins, revealed that stiff matrices increased nuclear accumulation of SP1 compared with soft matrices, indicating enhanced transcriptional regulatory activity of SP1 (Figure 8E,F). A comparable effect was observed following Yoda1 treatment (Figure 8G,H). Pharmacological inhibition of SP1 using Plicamycin/Mithramycin A (Mith) significantly decreased SP1 expression levels in MSCs (Figure 8I,J). Both real‐time quantitative polymerase chain reaction (RT‐qPCR) and WB analyses indicated that Mith treatment reduced STC2 expression (Figure 8I–K). To further substantiate the role of SP1, small interfering RNA (siRNA)‐mediated knockdown of SP1 was performed. Reanalysis of a published dataset (GSE165771) provided supportive evidence that *SP1* siRNA effectively downregulated *STC2* expression in HEK293 cells (Figure 8L). The knockdown efficiency of three distinct *Sp1* siRNAs was first evaluated by qPCR. Two siRNA sequences (si‐*Sp1*‐2 and si‐*Sp1*‐3), which significantly downregulated *Sp1* mRNA levels, were selected for subsequent functional assays (Figure 8M). Consistent with the pharmacological inhibition of SP1 by Mith, *Sp1* siRNA knockdown also resulted in a significant reduction in STC2 expression at both the mRNA and protein levels (Figure 8N–P).

Predictive analysis identified the consensus DNA‐binding motif recognized by SP1, as depicted in Figure 8Q. To assess SP1‐dependent *Stc2* promoter activity, luciferase reporter vectors containing either the WT or mutant (MUT) *Stc2* promoter sequence were constructed (Figure 8R). Dual‐luciferase reporter assays demonstrated that the PIEZO1 agonist Yoda1 enhanced the transcriptional activity of the WT *Stc2* promoter, whereas mutation of the SP1 binding sites in the *Stc2* promoter nullified the Yoda1‐induced activation (Figure 8S). Additionally, pharmacological inhibition of SP1 with Mith markedly suppressed Yoda1‐mediated upregulation of *Stc2* promoter activity (Figure 8T). Chromatin immunoprecipitation (ChIP) assays supported SP1 occupancy at the predicted *Stc2* promoter region in MSCs. Compared with the faint non‐specific band in the IgG negative control, the target DNA fragment exhibited a markedly stronger amplification band in the anti‐SP1 immunoprecipitate. Input chromatin was included as the positive amplification control (Figure 8U,V).

### 2.13 Calcium/Calmodulin‐Dependent Protein Kinase II (CaMKII) Mediates SP1 Activation via Ca^2^^+^ Signaling

Phosphorylation at the Thr453 residue has been proposed as a potential mechanism mediating SP1 activation [16]. WB results showed that Yoda1 treatment elevated phosphorylated SP1 (p‐SP1) levels (Figure S8A,B). Kinase prediction using the KinasePhos 3.0 tool identified CaMKII as a candidate kinase responsible for phosphorylating SP1 at Thr453 (Figure S8C). Yoda1 promoted CaMKII phosphorylation at threonine 286 (Thr286) in MSCs (Figure S8D,E). Subsequently, we examined whether Yoda1‐induced SP1 activation was dependent on Ca^2^^+^/CaMKII signaling. MSCs were treated with Yoda1 in combination with either the calcium chelator BAPTA‐AM (BAPTA) or the CaMKII inhibitor KN‐93. FCM analysis of intracellular Ca²⁺ levels revealed that Yoda1 strongly enhanced Ca^2^^+^ influx, whereas co‐treatment with BAPTA partially mitigated this effect (Figure S8F). Treatment with either BAPTA or KN‐93 decreased both nuclear SP1 levels (Figure S8G,H) and total cellular levels of p‐SP1, p‐CaMKII, and STC2 (Figure S8I–K).

### 2.14 Inhibition of SP1 Suppresses Osteogenesis and Facilitates Adipogenesis of MSCs Under PIEZO1 Activation

To investigate whether SP1 inhibition could counteract the lineage‐directing effects of PIEZO1 activation, the osteogenic and adipogenic capacities of MSCs were assessed under two distinct conditions: culture on stiff matrices or culture on soft matrices supplemented with Yoda1. In both PIEZO1‐activated conditions, Mith treatment attenuated osteogenic commitment of MSCs, evidenced by reduced mineralized nodule formation (Figure S9A,B), along with lowered ALP enzyme activity (Figure S9C,D). In contrast, under adipogenic induction conditions, ORO staining revealed that Mith increased the number and size of lipid droplets (Figure S9E,F). Furthermore, upon exposure of MSCs to bipotential inductive cues, Mith simultaneously restrained osteogenic specification and augmented the adipogenic tendency (Figure S9G). Altogether, these results showed that pharmacological inhibition of SP1 is sufficient to suppress MSC osteogenic differentiation and promote adipogenic commitment when PIEZO1 is activated.

## 3 Discussion

Investigations into the mechanical regulation of cell and tissue functions can provide theoretical insights for the development of clinically applicable biomimetic scaffolds for bone tissue regeneration [17]. As the primary load‐bearing structure responsible for supporting body weight, bone is subject to continuous mechanical stimulation, which is crucial for maintaining its homeostatic balance and regenerative capacity following injury [18]. A well‐documented example of this phenomenon is the impaired bone formation observed in astronauts exposed to microgravity conditions, which is reversible upon re‐exposure to terrestrial mechanical loading [19]. The mechanical confinement imposed by the ECM modulates cell volume, morphology, and protrusion dynamics, thereby orchestrating a diverse array of cellular processes, including differentiation, migration, proliferation, and apoptosis. Moreover, cell‐matrix interactions are inherently dynamic due to continuous matrix remodeling [20]. On stiff matrices, integrin‐mediated focal adhesions drive actin stress fiber assembly and elevate cellular contractility. This cytoskeletal tension is mechanically coupled to PIEZO1 at the membrane‐cytoskeleton interface, lowering its activation threshold to promote channel opening and calcium influx [21]. Based on these mechanobiological principles, the present study specifically investigated the influence of matrix stiffness, a critical mechanical property of the ECM, on MSC lineage commitment. Our findings demonstrated that stiff matrices enhanced osteogenic differentiation while suppressing adipogenic differentiation in MSCs, which is consistent with phenotypic observations documented in previous studies [22]. Extending previous research, our study further elucidated the activation of the PIEZO1/SP1/STC2 pathway in stiff matrix‐induced osteogenesis, which represents a new mechanistic insight that advances existing knowledge of PIEZO1‐regulated bone mechanobiology.

PIEZO1, a critical mechanosensitive ion channel, has been well documented to orchestrate stem/progenitor cell lineage specification. Importantly, the depletion of PIEZO1 rather than PIEZO2 in *Prrx1*‐lineage cells results in impaired bone formation and increased bone resorption. Both FSS and stiff matrices have been shown to augment the osteogenic differentiation of MSCs in vitro by activating PIEZO1 and facilitating Ca^2^^+^ influx [11]. Moreover, *Piezo1* deficiency in MSCs induces osteoclastogenesis, whereas its ablation in osteoclasts does not replicate this phenotype [23]. In periosteal progenitor cells, PIEZO1 activation drives osteogenic commitment, while *Piezo1* deficiency favors tendon regeneration [24]. Furthermore, PIEZO1 activation inhibits adipogenesis in fibrogenic/adipogenic progenitors in vitro and mitigates muscular fatty infiltration following rotator cuff tear [25]. Collectively, these studies establish PIEZO1 as a central mechanosensor regulating mesenchymal lineage allocation. In accordance with this paradigm, our study demonstrated that the opening of the PIEZO1 ion channel induced by stiff matrices enhanced osteogenesis and suppressed adipogenesis of MSCs, as corroborated by both in vitro and in vivo models. The downstream effectors of PIEZO1 include TFs such as Krüppel‐like factor 4 (KLF4) and activator protein‐1 (AP‐1) [25, 26], with nuclear translocation of SP1 observed in Yoda1‐stimulated macrophages [27]. In this study, scRNA‐seq reanalysis and nuclear‐cytoplasmic fractionation assays were employed to identify SP1 as a downstream TF of PIEZO1, and to elucidate its role in modulating MSC lineage commitment in response to stiff matrices, consistent with prior reports [28].

The precise balance of MSC lineage commitment is intricately associated with bone formation and metabolic homeostasis. MSC differentiation is tightly regulated by a multitude of critical mechanisms, including cell signaling pathways, hormonal modulation, and transcriptional regulation of target genes [29, 30]. Notably, the osteogenic differentiation of MSCs is orchestrated by a specialized transcriptional network consisting of pro‐osteogenic and anti‐adipogenic TFs [29]. Disruption of this balance, as seen with the deletion of regulators such as peroxisome proliferator‐activated receptor γ coactivator 1α (PGC‐1α) or insulin‐like growth factor 1 (IGF‐1), leads to abnormal MSC fate determination. This lineage shift manifests as diminished bone mass and augmented bone marrow adipose tissue (BMAT), which constitute hallmark pathological features of osteoporosis and skeletal aging [31, 32]. The present study also discovered that matrix stiffness‐activated PIEZO1 plays a role in regulating MSC differentiation.

Bone secretory proteins, referred to as osteokines, play a pivotal role in maintaining the homeostasis of bone and other organs [33]. STC2, a well‐characterized osteokine, promotes osteogenic differentiation while concomitantly suppressing adipogenic differentiation of MSCs by activating the extracellular signal‐regulated kinase (ERK) signaling pathway [34, 35]. The current study functionally validated the dual role of STC2 in lineage specification. Notably, we identified a novel upstream regulatory cascade whereby STC2 is transcriptionally activated by the PIEZO1/SP1 mechanosignaling axis. We further validated that STC2 promotes osteogenesis and suppresses adipogenesis independently of PIEZO1 activation, indicating dual mechanosensitive and autonomous roles in MSC fate control. Moreover, STC2 is known to enhance MSC survival by alleviating oxidative stress [36]. In this study, CCK‐8 assays revealed that STC2 improved MSC viability at several concentrations, with the strongest response observed around 500 ng/mL. Nevertheless, further investigations into cell proliferation and apoptosis were not conducted within the scope of this study. Furthermore, bone‐resident MSCs represent a principal source of osteokines. Three independent secretome profiling analyses have consistently identified STC2 as a secreted product of human bone marrow MSCs [13, 14, 15]. Prior research has demonstrated that MSC‐derived STC2 can modulate the functional phenotype of immune cells within inflammatory microenvironments [36, 37].

While this study provides valuable insights, several limitations warrant explicit acknowledgment. First, the findings are primarily based on static two‐dimensional (2D) in vitro culture systems, which are insufficient to fully recapitulate the dynamic three‐dimensional (3D) mechanical microenvironment characteristic of native bone. Second, this study did not account for other crucial biophysical attributes of the ECM, such as viscoelasticity, which is well‐documented to influence cellular behavior [38]. Third, the study did not investigate the impact of matrix stiffness on other functional cell populations within the bone microenvironment, particularly osteoclasts [39]. Fourth, the bone defect analyses in this study were conducted only at 7 days post‐surgery, reflecting early cell recruitment and initial mineralization but not supporting conclusions on mature bone regeneration.

In conclusion, the current study demonstrated that stiff matrices enhanced osteogenic differentiation and suppressed adipogenic differentiation of MSCs by activating the PIEZO1/SP1/STC2 pathway. STC2 also exerts direct pro‐osteogenic and anti‐adipogenic effects independently of PIEZO1 activation (Figure 8W). Furthermore, these findings pinpoint specific molecular targets that directly inform the rational design of stiffness‐optimized biomaterials conducive to bone regeneration.

## 4 Materials and Methods

### 4.1 scRNA‐seq Reanalysis

A previously published scRNA‐seq dataset (GSE166824), comprising human MSCs cultured on hydrogel matrices with two distinct stiffness levels (2 and 25 kPa) but lacking sample‐level biological replicates, was reanalyzed to assess the osteogenic and adipogenic differentiation potential of MSCs [22]. Following rigorous quality control, 1460 high‐quality single cells expressing 19824 genes were retained for subsequent analysis. Cell cluster annotation was performed utilizing the FindAllMarkers function within the Seurat package, with reference to the CellMarker database. TF activities were quantified using the decoupleR package. To evaluate MSC osteogenic and adipogenic differentiation, signature scores were calculated with the AUCell package based on established marker gene sets (osteogenic markers: *ACTA2*, *COL1A1*, *COL1A2*, *COMP*, *CREB3L1*, *SPP1*, *TAGLN*, *TMEM119*, *TNFRSF11B*, *WNT5B*; adipogenic markers: *ADIPOQ*, *APOE*, *CEBPA*, *CEBPB*, *FABP4*, *FABP5*, *LPL*, *PLIN1*, *PLIN4*, *PPARG*). GO and KEGG pathway enrichment analyses were conducted to characterize the functional signatures associated with distinct cell clusters. Cell differentiation potential was evaluated using the CytoTRACE2 package, while cell developmental trajectories were reconstructed using the Monocle3 package to delineate lineage progression patterns.

Furthermore, several publicly available bulk RNA‐seq datasets were retrieved from the NCBI Gene Expression Omnibus (GEO) database (GSE139121 [11], GSE181512 [40], GSE79814 [41], GSE113253 [29], and GSE165771 [42]) for comparative analysis. Detailed characteristics of each dataset are summarized in Table S1.

### 4.2 Culture and Identification of Mouse MSCs

Mouse MSCs were isolated and cultured following a previously established protocol [43]. Briefly, following euthanasia, femoral shafts were carefully dissected and minced into small fragments. The bone fragments underwent digestion with 0.2% collagenase type II (Solarbio, Beijing, China) for 15 min at 37°C. The digested bone fragments were seeded and cultured in α‐minimum essential medium (α‐MEM; Biosharp, Hefei, China) supplemented with 10% fetal bovine serum (FBS; Gibco, Grand Island, NY) and 1% penicillin/streptomycin (Servicebio, Wuhan, China), and maintained in a humidified incubator at 37°C with 5% CO_2_. Upon reaching 90% confluence, MSCs were harvested using 0.25% trypsin‐EDTA (Gibco) for subculture.

Characterization of mouse MSCs was conducted in accordance with the criteria recommended by the International Society for Cellular Therapy (ISCT) [44]. The expression of positive (CD73, CD90, and CD105) and negative (CD31, CD34, and CD45) surface markers was analyzed by FCM using a BD Accuri C6 Plus Flow Cytometer (BD, Franklin Lakes, NJ). The antibodies employed for FCM analysis are detailed in Table S2. Additionally, the multipotency of MSCs was verified by in vitro differentiation into osteogenic, adipogenic, and chondrogenic lineages, as evidenced by ARS (OriCell, Guangzhou, China), ORO (OriCell), and AB (OriCell) staining, respectively.

### 4.3 Osteogenic and Adipogenic Differentiation Assays of MSCs

Osteogenic differentiation was induced using the mouse MSC osteogenic induction medium (OriCell). After 21 days of induction, MSCs were fixed with 4% paraformaldehyde (PFA), stained with ARS solution for 20 min to visualize osteogenic mineralization at room temperature, and imaged by microscopy (Olympus, Tokyo, Japan). For quantitative analysis, the bound ARS was solubilized in 10% acetic acid, and the absorbance at 405 nm (A405) was measured using a microplate reader (PerkinElmer, Waltham, MA) [45]. ALP staining was conducted after 7 days of osteogenic induction utilizing a BCIP/NBT Alkaline Phosphatase Color Development Kit (Beyotime, Shanghai, China). ALP enzymatic activity was quantified employing an Alkaline Phosphatase Assay Kit (Beyotime). The protein expression levels of RUNX2, ALP, and OCN were assessed by WB analysis following 7 days of osteogenic induction.

Adipogenic differentiation was induced using the mouse MSC adipogenic induction medium (OriCell). After 7 days, MSCs were fixed and stained with ORO working solution for 10 min at room temperature to visualize lipid droplets. Lipid droplet area was quantified using ImageJ software, with values expressed relative to the control group. Concurrently, after 7 days of adipogenic induction, MSCs were harvested by trypsinization, fixed with 4% PFA, stained with Nile Red solution (Beyotime), and analyzed by FCM. For fluorescence imaging, adherent cells were co‐stained with Nile Red and 4',6‐diamidino‐2‐phenylindole (DAPI) (Beyotime) for 20 min at room temperature in the dark. The protein expression levels of PPARγ and FABP4 were detected by WB analysis after 5 days of adipogenic induction.

The bipotential induction medium was prepared by mixing osteogenic and adipogenic induction media as previously reported [22]. After 7 days of bipotential induction, MSCs were fixed and subjected to successive ALP and ORO staining.

### 4.4 Treatment of Mouse MSCs

MSCs were cultured on type I collagen‐coated polyacrylamide gels (Petrisoft; Matrigen, West Lafayette, IN) with elastic moduli of 2 kPa (soft matrix) and 25 kPa (stiff matrix). F‐actin cytoskeleton organization of MSCs was visualized using the fluorescent probe phalloidin‐Alexa Fluor 594 (Beyotime). PIEZO1 expression was assessed by WB analysis. MSCs cultured on stiff matrices were treated with GsMTx4 (1 µM; MCE, Shanghai, China), a peptide inhibitor of multiple mechanosensitive ion channels, to suppress PIEZO1 activity. Complementary rescue assays were conducted to validate the underlying molecular signaling pathway, in which MSCs cultured on polyacrylamide gels were treated with recombinant mouse STC2 (500 ng/mL; MCE), the PIEZO1 agonist Yoda1 (2 µM; MCE), or the SP1 inhibitor Mith (500 nM; MCE).

In a separate experimental setup, MSCs were cultured on conventional tissue culture polystyrene (TCPS) plates and exposed to Yoda1 (2 µM) in the presence or absence of its competitive antagonist Dooku1 (7.5 µM; MCE). Additionally, MSCs were treated with gradient concentrations of recombinant STC2 (100, 500 ng/mL), Mith (100, 500 nM), the intracellular calcium chelator BAPTA‐AM (20 µM; MCE), or the CaMKII inhibitor KN‐93 (1 µM; MCE).

Under bipotential induction conditions, MSCs treated with STC2 or vehicle were collected for bulk transcriptome profiling by RNA‐seq.

### 4.5 Prediction of Transcription Factors Regulating Stc2 Expression and Their Upstream Kinases

The mouse *Stc2* promoter sequence was obtained from the NCBI database. Potential TFs capable of regulating *Stc2* were predicted using four bioinformatics resources, namely JASPAR 2016 (https://jaspar.elixir.no/), ALGGEN‐PROMO (https://alggen.lsi.upc.es/cgi‐bin/promo_v3/promo/promoinit.cgi?dirDB=TF_8.3), AnimalTFDB version 4.0 (https://guolab.wchscu.cn/AnimalTFDB4//#/), and ChIP‐Atlas (https://chip‐atlas.org/). DEGs were subjected to TF target enrichment analysis using the TRRUST version 2 database (https://www.grnpedia.org/trrust/). Key upstream TFs were identified according to the statistical significance of target gene enrichment among the DEGs.

To identify candidate upstream kinases responsible for SP1 phosphorylation at Thr453, the KinasePhos 3.0 software was employed [46].

### 4.6 Dual‐Luciferase Reporter Assays

Dual‐luciferase reporter constructs were generated by cloning either the WT or MUT promoter sequences of the *Stc2* gene into the pGL4.10 vector (Promega, Madison, WI). To assess the functional regulation of the *Stc2* promoter by transcription factor SP1, HEK293T cells were co‐transfected with the firefly luciferase reporter construct and the Renilla luciferase internal control plasmid. The cells were then treated with Yoda1 or Mith. At 48 h post‐transfection, cells were harvested and lysed, and the activities of firefly and Renilla luciferase were measured using the Dual‐Luciferase Reporter Assay System (Promega).

### 4.7 ChIP Assays

ChIP was performed using a commercial kit (BersinBio, Guangzhou, China) following the protocol provided by the manufacturer. Briefly, MSCs were cross‐linked with 1% formaldehyde, quenched with glycine, lysed, and sonicated to generate 200–600 bp chromatin fragments. Pre‐cleared chromatin was incubated overnight at 4°C with a ChIP‐grade anti‐SP1 antibody, with normal rabbit IgG as the negative control. Antibody‐chromatin complexes were captured with protein A/G beads, washed, eluted, and reverse cross‐linked at 65°C overnight. Following RNase A and proteinase K digestion, DNA was purified via phenol‐chloroform extraction. The *Stc2* promoter region was amplified by PCR, and the products were resolved on a GelRed‐containing agarose gel, with input chromatin as the positive control. Primer sequences for ChIP‐PCR are provided in Table S5.

### 4.8 Fabrication and Physical Characterization of GelMA Hydrogels

GelMA with a methacrylation degree of 30% (denoted as GelMA‐30) was procured from Engineering For Life (EFL, Suzhou, China). Hydrogel precursor solutions were prepared by dissolving GelMA at concentrations of 5%, 7.5%, 10%, 12.5%, 15%, and 17.5% (w/v) in phosphate‐buffered saline (PBS) containing 0.25% (w/v) of the photoinitiator lithium phenyl(2,4,6‐trimethylbenzoyl)phosphinate (LAP; EFL). Hydrogels were subsequently fabricated by exposing the precursor solutions to ultraviolet (UV) light (EFL) at an intensity of 25 mW/cm^2^ for 30 s. For the characterization of mechanical properties, GelMA hydrogel samples were subjected to unconfined uniaxial compression testing by means of the ElectroForce 3200 Series (TA Instruments, New Castle, DE). The compression tests were conducted at a compressive rate of 1 mm/min to a maximum strain of 50%. The compressive modulus was calculated through linear regression analysis of the stress–strain curves.

### 4.9 Mice

*Prrx1^CreERT2^* (NM‐KI‐225017; Shanghai Model Organisms Center), *Piezo1^f/f^* mice (#029213; Jackson Laboratory), and *Rosa26^mTmG^* mice (C001192; Cyagen Biosciences) were employed in this study. *Prrx1*‐expressing cells function as MSCs in the bone, white adipose tissue, and dermis of adult mice [47]. To generate iCKO models targeting PIEZO1 in mesenchymal lineages, *Piezo1^f/f^* mice were crossbred with *Prrx1^CreERT2^* mice, producing *Prrx1^CreERT2^;Piezo1^f/f^* mouse lines. These mice were subsequently crossbred with *Rosa26^mTmG^* reporter mice to establish triple‐transgenic *Prrx1^CreERT2^;Piezo1^f/f^;Rosa26^mTmG^* mouse lines. *Prrx1^CreERT2^* or *Prrx1^CreERT2^;Rosa26^mTmG^* mouse lines served as the respective controls in all experiments to minimize the potential influence of genetic background. All experimental animals were maintained on a C57BL/6J genetic background and housed in specific pathogen‐free (SPF) animal facilities. TAM (Sigma‐Aldrich) was administered via intraperitoneal injection at a dosage of 75 mg/kg once daily for 1 week before surgery to achieve the specific activation of Cre recombinase. Both control and experimental groups received the same TAM treatment regimen. Genotyping analysis of all mouse lines was performed by PCR using specific primer pairs, as detailed in Table S6. For in vitro experiments, MSCs were isolated from the femurs of *Prrx1*‐lineage‐specific *Piezo1* iCKO mice and their corresponding control mice to assess their osteogenic and adipogenic differentiation potential. In the cellular assays, 4‐OHT (100 nM; MCE) was utilized to activate Cre recombinase in primary MSCs.

### 4.10 Mouse Femoral Bone Defect and Hindlimb Unloading Models

All animal procedures received approval from the Animal Research Committee of Hebei Medical University (Shijiazhuang, China; Ethics Approval No. IACUC‐Hebmu‐2025130), and were performed in strict accordance with the 3Rs (Replacement, Reduction, Refinement) principles. Twelve‐week‐old WT C57BL/6J mice were procured from Beijing SPF Biotechnology Co., Ltd. Following isoflurane inhalation anesthesia and surgical site depilation, a longitudinal incision was made to expose the distal femur. A unicortical bone defect with a diameter of 1 mm was created approximately 1 mm proximal to the growth plate in the distal femur using a sterile dental drill [48]. The EPLQLKM (H‐Glu‐Pro‐Leu‐Gln‐Leu‐Lys‐Met‐OH) peptide (1 mg/mL; MCE), a previously validated MSC‐specific affinity peptide [49], was incorporated into GelMA solutions to facilitate the targeted recruitment of endogenous MSCs to the bone defect site. Sterile 5% GelMA, 15% GelMA, or 5% GelMA supplemented with 500 ng/mL STC2 was injected into the bone defect, followed by in situ photocrosslinking using UV exposure for 30 s. At 7 days post‐surgery, the mice were euthanized for the collection of femoral samples.

To simulate mechanical unloading conditions, the HLU protocol was initiated 24 h after bone defect surgery exclusively in WT mice rather than in genetically modified mice [50]. Briefly, mice were suspended at a 30° angle to ensure their hindlimbs remained off the ground. The mice had unrestricted access to food and water throughout the experiment. The general appearance, eating behavior, and tail condition of the mice were monitored daily to assess their health status. The HLU period was maintained for 6 consecutive days post‐surgery.

### 4.11 µCT Scanning and Analysis

Femoral samples were scanned using a SkyScan 1176 µCT scanner (Bruker, Billerica, MA) at a resolution of 9 µm/pixel. Following scanning, three‐dimensional (3D) images of the samples were reconstructed using NRecon software (version 1.6; Bruker). The bone microstructure within the defect region was quantitatively analyzed with CTAn software (version 1.9; Bruker). The following parameters were quantified: BV/TV, trabecular thickness (Tb.Th), Tb.N, and Tb.Sp. Coronal and sagittal plane images of the bone defects were generated using CTVox software (version 2.0; Bruker).

### 4.12 Histological Analysis

The paraffin‐embedded mouse femoral samples were cut into sagittal sections with a thickness of 4 µm. HE staining was performed to evaluate the general histological morphology of the bone defect region. Masson's trichrome staining was employed to assess collagen deposition.

CD73 serves as a canonical marker for MSCs residing in the bone marrow niche [51]. IFstaining was conducted to detect the expression levels of PIEZO1, STC2, and OSX in CD73‐positive cells. In brief, paraffin sections were deparaffinized and rehydrated. Antigen retrieval was achieved in EDTA buffer (pH 8.0; Solarbio) for 15 min at 95°C. Subsequently, the sections were blocked with 20% donkey serum (Solarbio) containing 0.5% Triton X‐100 (Biosharp) for 1 h at room temperature, and then incubated with primary antibodies overnight at 4°C. After washing, the sections were incubated with fluorophore‐conjugated secondary antibodies for 1 h at room temperature, and counterstained with DAPI or propidium iodide (PI; Beyotime) for 15 min to label cell nuclei. The fluorescence intensity was quantified by ImageJ software. The fluorescence intensity or count of target‐positive cells was calculated and expressed relative to the control group.

### 4.13 Statistical Analysis

For in vitro experiments using MSCs, primary mouse cells were obtained from different animals in five independent experiments. Continuous variables were expressed as mean ± standard deviation (SD), and statistical analyses were performed using GraphPad Prism software (version 9.0; San Diego, CA). All statistical tests were two‐tailed. Unpaired Student's *t*‐test was employed for comparisons between two groups. For comparisons involving three or more experimental groups, one‐way analysis of variance (ANOVA) was performed, followed by post‐hoc multiple comparison tests: Tukey's test for all pairwise comparisons among groups, and Šídák's test for pre‐specified comparisons of each treatment group versus the control group. *p* < 0.05 was considered statistically significant.

## Author Contributions

**Yingze Zhang**, **Yanbin Zhu**, and **Wei Chen** designed the study. **Shuo Zhang**, **Siteng Li**, **Wenzhong Chen**, and **Qingcheng Song** performed the experiments. Shuo Zhang, **Haiyue Zhao**, **Peng Wang**, and **Yiran Zhang** analyzed the data and prepared the figures. Shuo Zhang, **Wenquan Liang**, and **Shaowei Zheng** wrote the manuscript. **Juan Wang** and Yanbin Zhu revised the manuscript. Yingze Zhang supervised the entire project. All authors reviewed and approved the final manuscript. Shuo Zhang, Siteng Li, and Wenzhong Chen contributed equally to this work.

## Funding

This work was supported by the National Natural Science Foundation of China (32130052, U23A6008, 82572721, 82402875), the China Postdoctoral Science Foundation (2025T180663, 2024M751530), and the Medical Scientific Research Foundation of Guangdong (A2024366).

## Conflicts of Interest

The authors declare no conflicts of interest.

## Ethics Statement

All animal experiments were performed in strict accordance with the guidelines of the Animal Research Committee of Hebei Medical University (Ethics Approval No. IACUC‐Hebmu‐2025130).

## Supporting information




**Supporting File**: advs77385‐sup‐0001‐SuppMat.pdf.

## Data Availability

The bulk RNA‐seq data from this study have been deposited in the NCBI GEO database under accession number GSE338005. All other data supporting the findings of this study are available from the corresponding authors upon reasonable request.
