# Supplementary material for: Matrix Stiffness Orchestrates Mesenchymal Stem Cell Lineage Commitment Toward Osteogenesis and Adipogenesis Through the PIEZO1/SP1/STC2 Axis
Source: Adv Sci (Weinh). 2026 Sep 6:e77385. Online ahead of print. doi: 10.1002/advs.77385 (PMC13547798; doi:10.1002/advs.77385)
Supplement: Supplementary file 1 [file ADVS-9999-e77385-s001.pdf]

SUPPORTING INFORMATION FOR

Matrix Stiffness Orchestrates Mesenchymal Stem Cell Lineage Commitment Toward Osteogenesis and Adipogenesis Through the PIEZO1/SP1/STC2 Axis

Shuo Zhang[1,2,#], Siteng Li[1,2,3,#], Wenzhong Chen[2,#], Qingcheng Song[2], Haiyue Zhao[2], Peng Wang[4], Yiran Zhang[5], Wenquan Liang[6], Shaowei Zheng[7], Juan Wang[2], Wei Chen[2,*], Yanbin Zhu[2,*], Yingze Zhang[1,2,*]

[1] School of Medicine, Nankai University, Tianjin 300071, China

[2] Department of Orthopedic Surgery, The Third Hospital of Hebei Medical University, Shijiazhuang 050051, Hebei, China

[3] Department of Orthopaedic Trauma, Centre for Orthopaedic Surgery, The Third Affiliated Hospital, Southern Medical University, Guangzhou 510515, Guangdong, China

[4] Department of Orthopedics, Fuzhou University Affiliated Provincial Hospital, Fuzhou 350002, Fujian, China

[5] Department of Hand and Foot Surgery, Shenzhen Second People's Hospital, Shenzhen 518035, Guangdong, China

[6] Department of Anatomy, School of Basic Medical Sciences, Southern Medical University, Guangzhou 510515, Guangdong, China

[7] Department of Sports Medicine and Rehabilitation, Shenzhen Xinhua Hospital, Peking University Shenzhen Hospital, Shenzhen 518131, Guangdong, China

[#] These authors contributed equally: Shuo Zhang, Siteng Li, Wenzhong Chen

***Correspondence to**

Yingze Zhang: School of Medicine, Nankai University, Tianjin 300071, China

Yanbin Zhu: Department of Orthopedic Surgery, The Third Hospital of Hebei Medical University, Shijiazhuang 050051, Hebei, China

Wei Chen: Department of Orthopedic Surgery, The Third Hospital of Hebei Medical University, Shijiazhuang 050051, Hebei, China

Materials and Methods

1. Cell Viability Assays

Cell viability was evaluated by Cell Counting Kit-8 (CCK-8; MCE) assays. Briefly, MSCs were seeded in 96-well plates at a density of 5×10^3 cells per well and subjected to the respective treatments. Thereafter, the culture medium was replaced with 100 μL of fresh Dulbecco's Modified Eagle's Medium/Ham's F-12 (DMEM/F-12; Biosharp) containing 10% (v/v) CCK-8 reagent per well. Following incubation for 2 h at 37 °C in the dark, the absorbance at 450 nm (A450) was measured using a microplate reader.

2. Western Blotting (WB) Analysis

After treatment, MSCs were lysed with radioimmunoprecipitation assay (RIPA) lysis buffer (Beyotime) supplemented with protease and phosphatase inhibitors (Servicebio). Protein samples were separated by SDS-PAGE and transferred onto polyvinylidene difluoride (PVDF) membranes (EMD Millipore, Billerica, MA). The membranes were blocked with 5% bovine serum albumin (BSA; Solarbio) for 1 h at room temperature, followed by incubation with primary antibodies overnight at 4 °C.

After washing, the membranes were incubated with horseradish peroxidase (HRP)-conjugated secondary antibodies for 1 h and visualized with enhanced chemiluminescence (ECL) detection reagent (Biosharp, Hefei, China). Band intensities were quantified using ImageJ software (NIH, Bethesda, MD). Target protein signals were normalized to GAPDH or Histone H3, and relative expression levels were presented as fold changes versus the control group. The antibodies are listed in Table S2.

3. Real-time Quantitative Polymerase Chain Reaction (RT-qPCR)

Following the indicated treatments, total RNA was isolated from MSCs using the RNA-easy Isolation Reagent (Vazyme, Nanjing, China). RNA concentration and purity were determined with a NanoDrop 2000 spectrophotometer. Complementary DNA (cDNA) was synthesized via reverse transcription with a cDNA Synthesis Kit (Yeasen, Shanghai, China). Quantitative PCR was performed using the SYBR Green Master Mix (Yeasen) on the LightCycler 480 Real-Time PCR System (Roche, Basel, Switzerland). The relative expression levels of target genes were normalized to the reference gene *Gapdh* and calculated using the comparative $2^{-\Delta\Delta Ct}$ method. Primer sequences are provided in Table S3.

4. RNA Interference Targeting *Sp1*

RNA interference (RNAi) mediates highly efficient and sequence-specific mRNA degradation, thereby downregulating the expression of the encoded target protein. MSCs were transfected with small interfering RNA (siRNA) using Lipofectamine 3000 reagent (Thermo Fisher Scientific, Waltham, MA) following the manufacturer's

protocol. The knockdown efficiency was assessed by RT-qPCR at 48 h post-transfection and by WB assays at 72 h post-transfection. The siRNA sequences targeting *Sp1* used in this study are listed in Table S4.

5. Flow Cytometric Measurement of Intracellular Ca^{2+} Signals

MSCs were harvested by trypsinization and subsequently incubated with the calcium-sensitive fluorescent probe Fluo-4 AM (Beyotime) at 2 μM in Hanks' Balanced Salt Solution (HBSS; Servicebio) for 20 min in the dark. The cells were divided into three treatment groups: (1) treatment with vehicle, (2) treatment with 2 μM Yoda1, and (3) pretreatment with 20 μM calcium chelator BAPTA-AM for 20 min, followed by stimulation with 2 μM Yoda1. Intracellular Ca^{2+} signals were assessed by FCM analysis based on the fluorescence intensity of Fluo-4. Data analysis was conducted using FlowJo software (version 10.10.0).

6. Immunohistochemical (IHC) Staining

IHC staining was performed to assess PPARγ expression within bone defect sites. After paraffin sections were deparaffinized and rehydrated, endogenous peroxidase activity was quenched with 3% H_2O_2. Antigen retrieval was conducted in EDTA buffer (pH 8.0) at 95 °C for 15 min. Sections were blocked with 20% donkey serum for 1 h and then incubated with rabbit anti-PPARγ primary antibody (1:200) or non-immune rabbit IgG (negative control) overnight at 4 °C. After washing, the sections were incubated with HRP-conjugated secondary antibody (Servicebio) for 1 h, visualized with diaminobenzidine (Servicebio), and counterstained with hematoxylin. The number of PPARγ-positive cells was quantified and expressed relative to the control group.

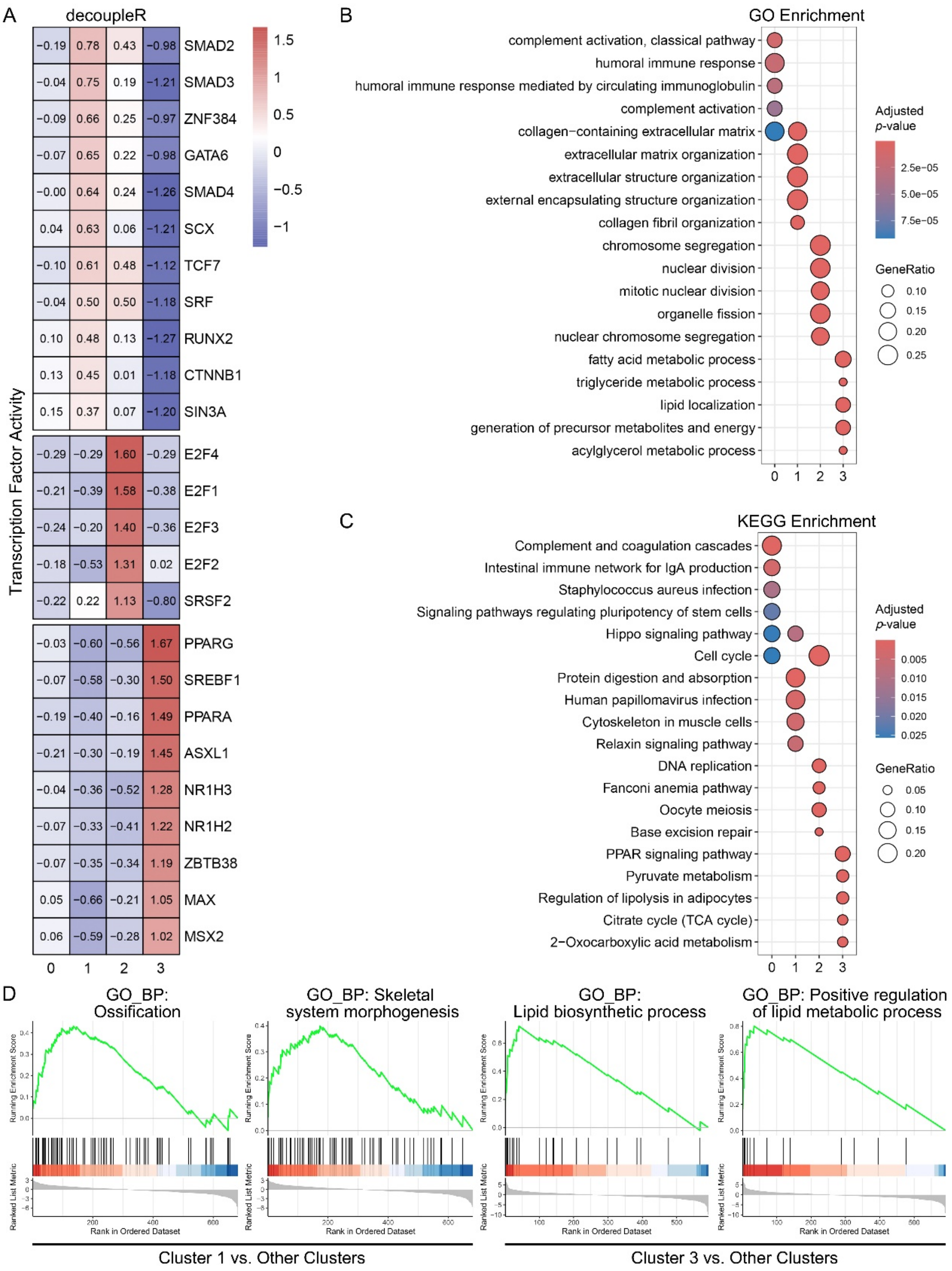

Figure S1: scRNA-seq reanalysis reveals the TF activity and functional enrichment patterns across distinct cell clusters

(A) TF activity assessed by decoupleR across clusters. (B) GO enrichment analysis. (C) KEGG pathway enrichment analysis. (D) GSEA plots depicting the enrichment of gene

sets related to osteogenesis or adipogenesis from scRNA-seq data.

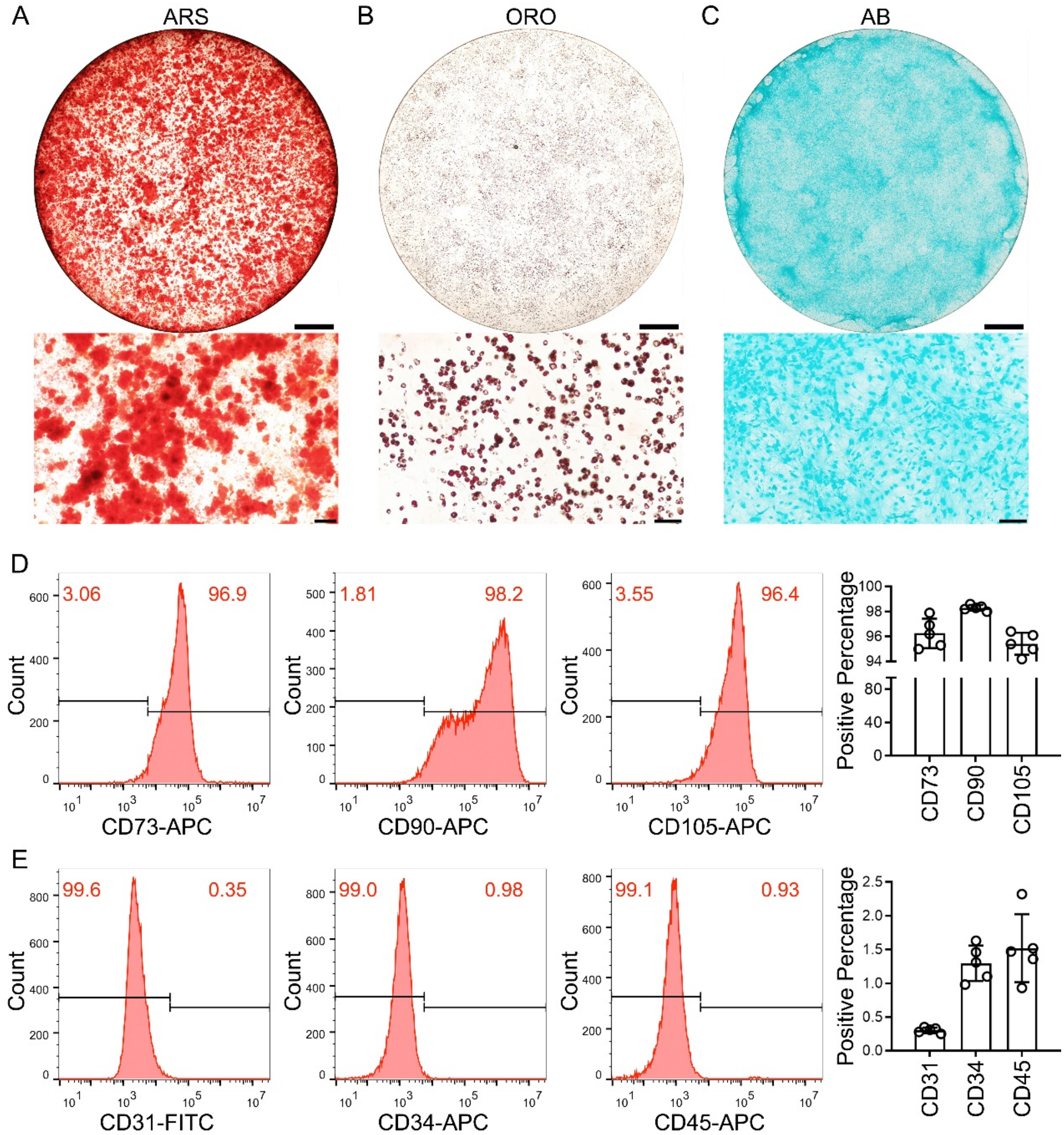

Figure S2: Characterization of murine MSCs derived from femoral tissues

(A–C) *In vitro* assessment of osteogenic, adipogenic, and chondrogenic differentiation of MSCs using ARS, ORO, and AB staining, respectively (A–C: upper scale bar, 2 mm; A: lower scale bar, 200 μm; B–C: lower scale bar, 100 μm). (D, E) Flow cytometry (FCM) analysis of surface markers CD73, CD90, CD105, CD31, CD34, and CD45 in MSCs. All experimental data are presented as mean ± SD. $n = 5$ biological replicates.

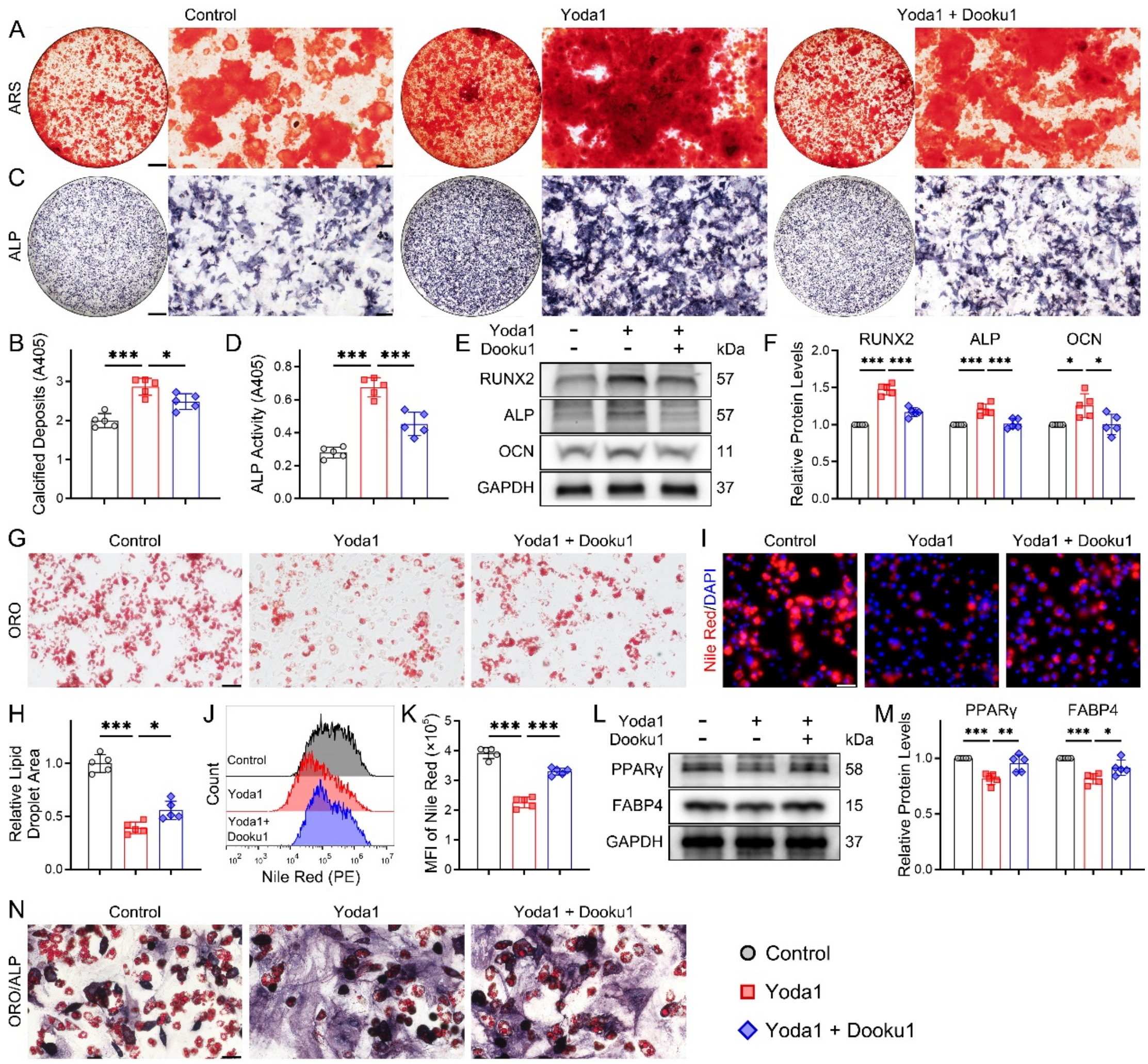

Figure S3: PIEZO1 agonist Yoda1 promotes osteogenesis and suppresses adipogenesis in MSCs

(A, B) ARS staining showing the calcified deposits of MSCs (left scale bar, 2 mm; right scale bar, 200 μm). (C, D) ALP staining and ALP activity assays of MSCs (left scale bar, 2 mm; right scale bar, 200 μm). (E, F) Representative WB images and statistical analysis of RUNX2, ALP, OCN, and GAPDH in MSCs. (G, H) ORO staining showing the lipid droplets of MSCs (scale bar, 100 μm). (I–K) Fluorescence images and FCM analysis of Nile Red staining of MSCs (scale bar, 50 μm). (L, M) Representative WB images and statistical analysis of PPARγ, FABP4, and GAPDH in MSCs. (N) ALP

staining and ORO staining evaluating the osteogenic and adipogenic differentiation potential of MSCs (scale bar, 50 μm). Cells were cultured on tissue culture polystyrene (TCPS) plates. All experimental data are presented as mean ± SD. *$p < 0.05$, **$p < 0.01$, ***$p < 0.001$. $n = 5$ biological replicates.

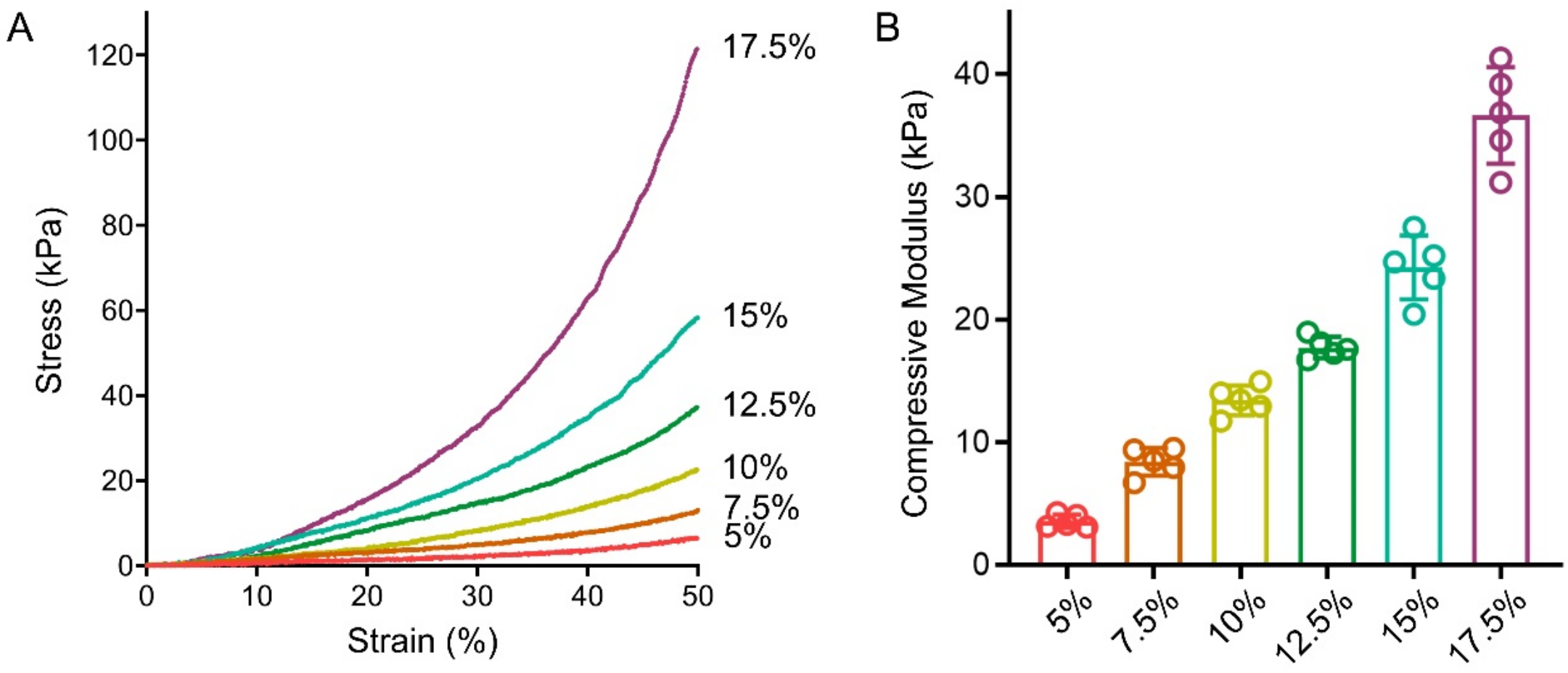

Figure S4: Compression tests of GelMA hydrogels at varying concentrations

(A) Representative compressive stress–strain curves of GelMA hydrogels. (B) Compressive modulus values of GelMA hydrogels. All experimental data are presented as mean ± SD. $n = 5$ independent experiments.

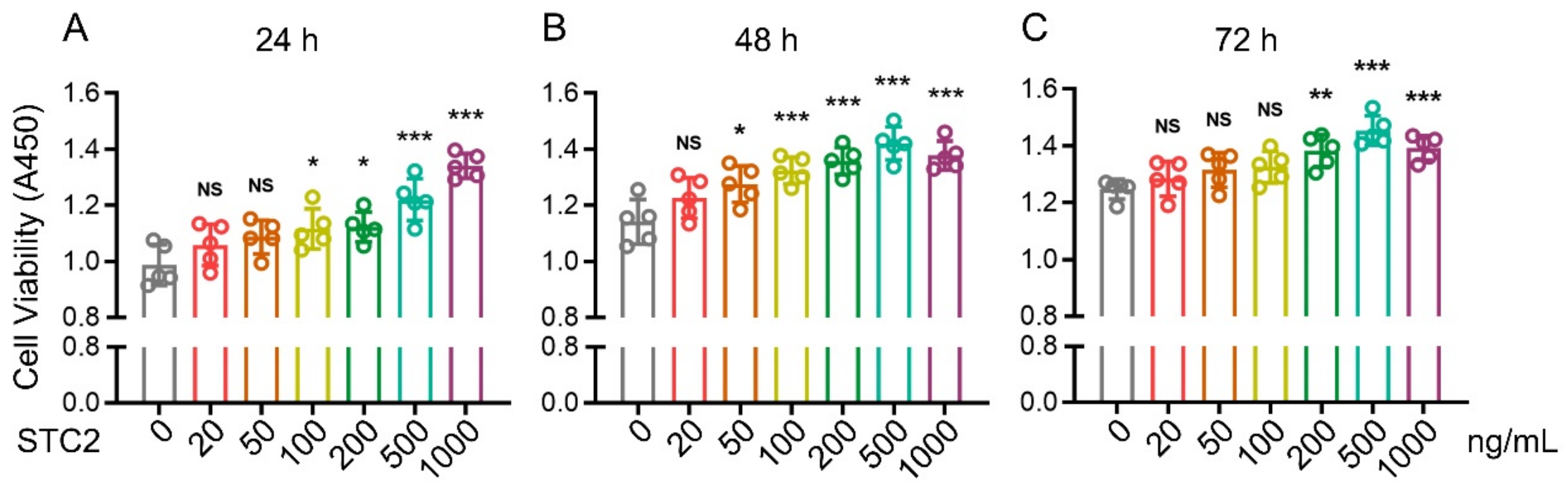

Figure S5: STC2 increases CCK-8 metabolic activity in MSCs

(A–C) MSCs were treated with serial concentrations of STC2 for 24, 48, and 72 h, and

cell viability was quantified using CCK-8 assays. All experimental data are presented as mean ± SD. *$p < 0.05$, **$p < 0.01$, ***$p < 0.001$, NS: not significant. $n = 5$ biological replicates.

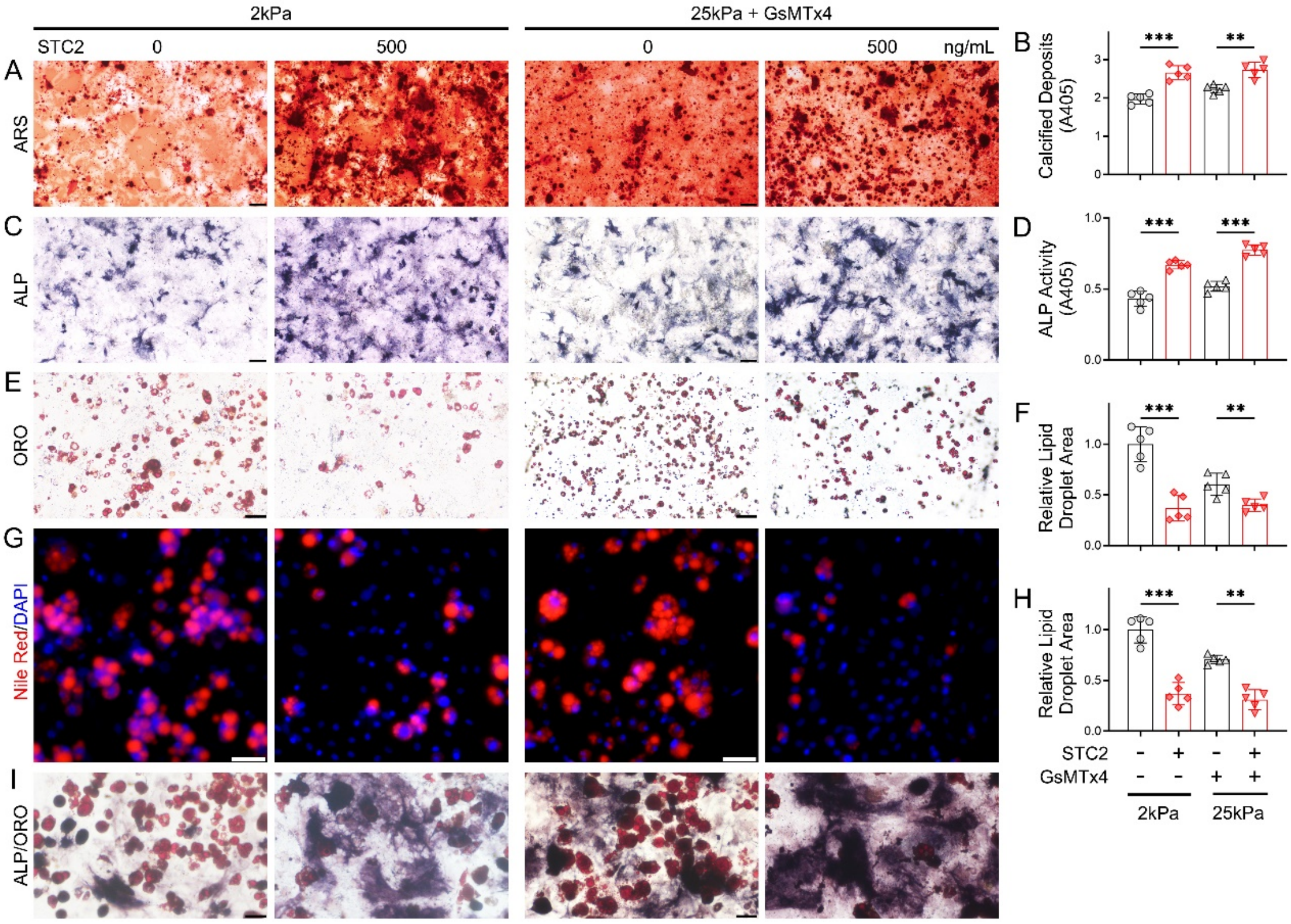

Figure S6: STC2 promotes osteogenesis and suppresses adipogenesis of MSCs under PIEZO1-inactivated conditions

(A, B) ARS staining showing the calcified deposits of MSCs (scale bar, 200 μm). (C, D) ALP staining and ALP activity assays of MSCs (scale bar, 200 μm). (E, F) ORO staining showing the lipid droplets of MSCs (scale bar, 100 μm). (G, H) Nile Red staining visualizing the lipid droplets of MSCs (scale bar, 50 μm). (I) ALP staining and ORO staining evaluating the osteogenic and adipogenic differentiation potential of MSCs (scale bar, 50 μm). Cells were cultured on type I collagen-coated polyacrylamide gels with elastic moduli of 2 kPa and 25 kPa. All experimental data are presented as

mean ± SD. *$p < 0.05$, **$p < 0.01$, ***$p < 0.001$. $n = 5$ biological replicates.

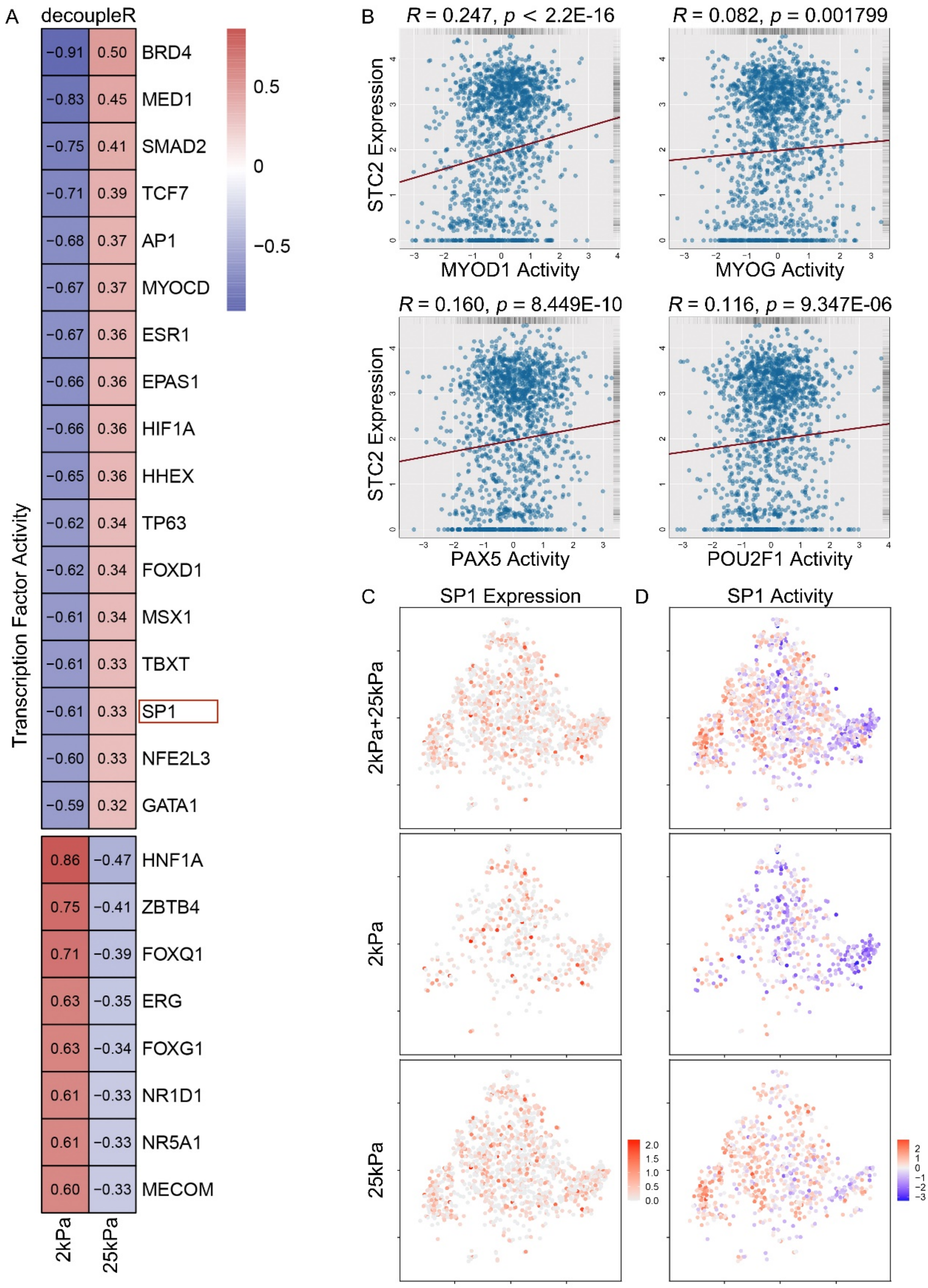

Figure S7: scRNA-seq reanalysis supports SP1 as a candidate stiffness-responsive transcription factor

(A) TF activity assessed by decoupleR under distinct stiffness culture conditions. (B) Cell-level exploratory correlation analysis of *STC2* expression and the activity of MYOD1, MYOG, PAX5, and POU2F1. (C) t-SNE plots illustrating *SP1* expression under distinct stiffness culture conditions. (D) t-SNE plots illustrating SP1 activity under distinct stiffness culture conditions.

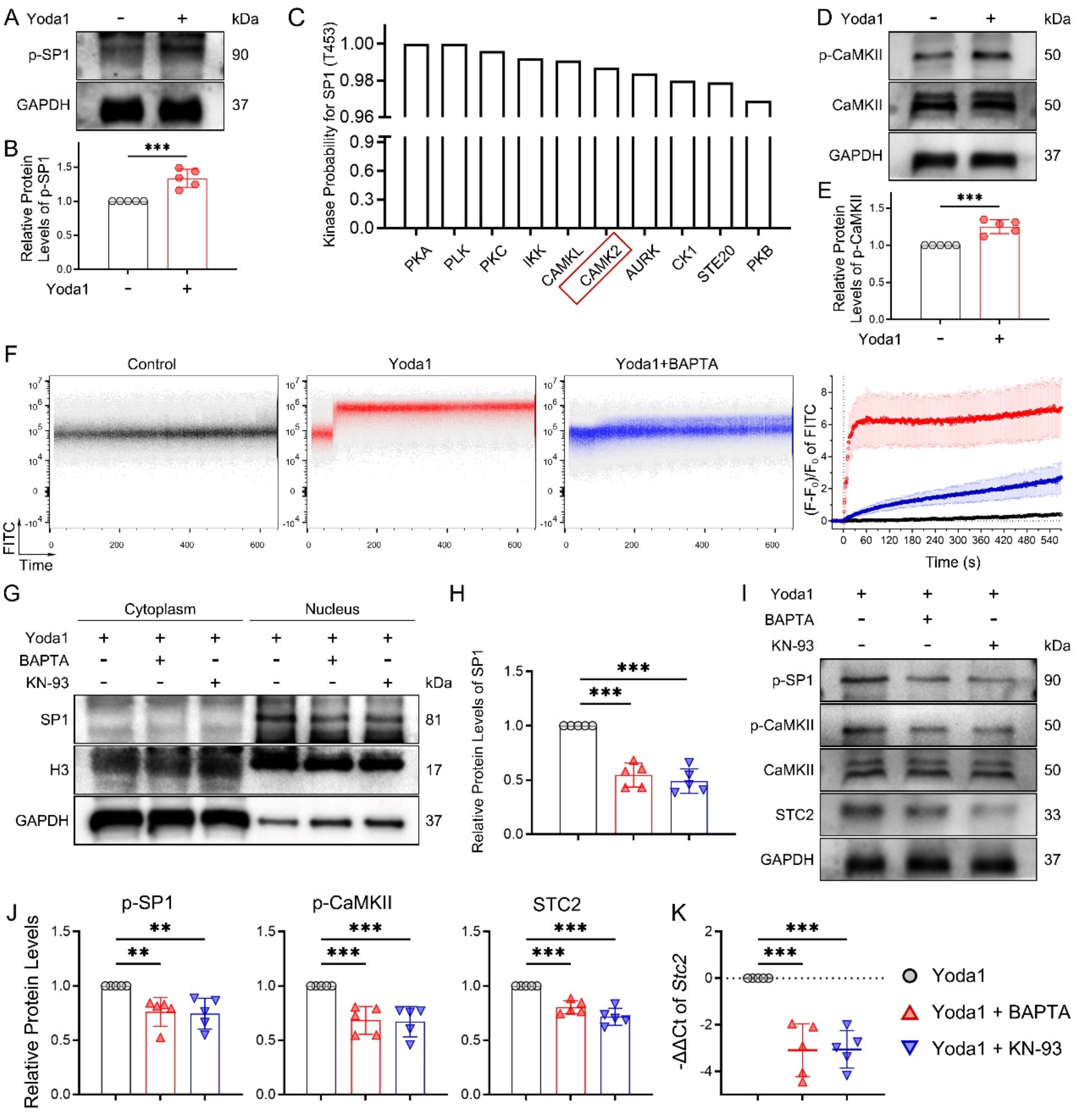

Figure S8: Ca²⁺/CaMKII signaling contributes to Yoda1-induced SP1 activation

(A, B) Representative WB images and statistical analysis of p-SP1 and GAPDH in

MSCs. (C) Prediction of potential kinases targeting SP1 at Thr453 using the KinasePhos 3.0 tool. (D, E) Representative WB images and statistical analysis of CaMKII, p-CaMKII, and GAPDH in MSCs. (F) FCM analysis of intracellular calcium levels in MSCs assessed by the Fluo-4 AM probe. (G, H) Representative WB images and statistical analysis of SP1, Histone H3, and GAPDH in MSCs. (I, J) Representative WB images and statistical analysis of p-SP1, p-CaMKII, CaMKII, STC2, and GAPDH in MSCs. (K) The mRNA levels of *Stc2* in MSCs. All experimental data are presented as mean ± SD. $*p < 0.05$, $**p < 0.01$, $***p < 0.001$. $n = 5$ biological replicates.

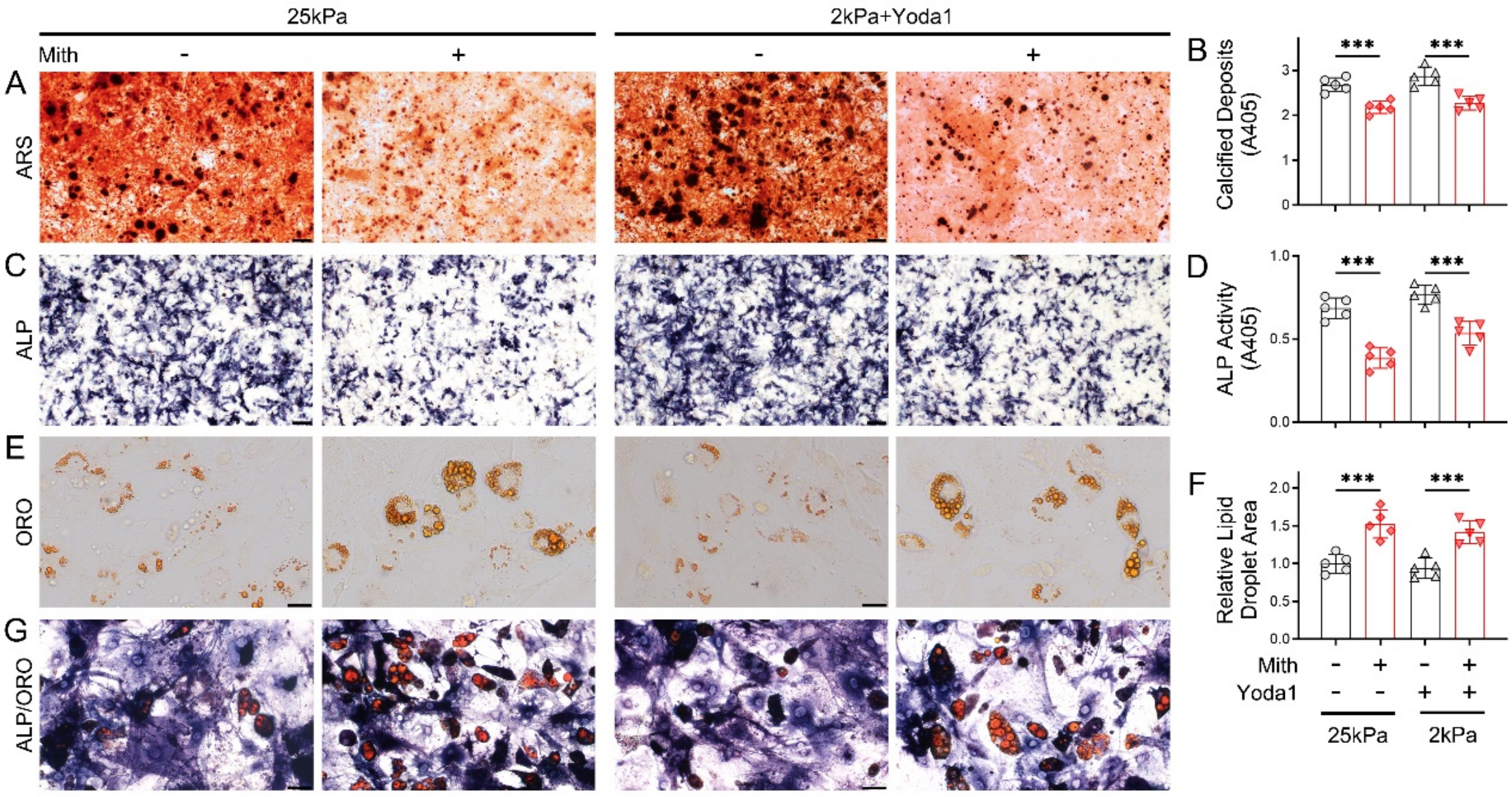

Figure S9: Inhibition of SP1 suppresses osteogenesis and facilitates adipogenesis of MSCs under PIEZO1 activation

(A, B) ARS staining showing the calcified deposits of MSCs (scale bar, 200 μm). (C, D) ALP staining and ALP activity assays of MSCs (scale bar, 200 μm). (E, F) ORO staining showing the lipid droplets of MSCs (scale bar, 25 μm). (G) ALP staining and ORO staining evaluating the osteogenic and adipogenic differentiation potential of

MSCs (scale bar, 50 μm). Cells were cultured on type I collagen-coated polyacrylamide gels with elastic moduli of 2 kPa and 25 kPa. All experimental data are presented as mean ± SD. *$p < 0.05$, **$p < 0.01$, ***$p < 0.001$. $n = 5$ biological replicates.

Table S1: Detailed Characteristics of GEO Datasets

Accession Number	Sequence Type	Group Information	Reference	Used in Figures
GSE166824	scRNA-seq	Human BM-MSCs cultured on soft (2 kPa) / stiff (25 kPa) matrices with osteogenic and adipogenic induction.	[22]	Figure 1A–K, 5A–C, 5M–P
GSE139121	bulk RNA-seq	Femur and humerus bone tissues from *Prrx1*^{Cre} / *Prrx1*^{Cre};*Piezo1*$^{f/f}$ mice at postnatal day 0 (P0). *n* = 2.	[11]	Figure 4A, 5F
GSE181512	bulk RNA-seq	Rat BM-MSCs 3D-cultured in soft (2 kPa) / stiff (18 kPa) hydrogels. *n* = 3.	[40]	Figure 5E
GSE79814	bulk RNA-seq	Mouse BM-MSCs: non-induced control, 7 d / 15 d osteogenic induction, 7 d adipogenic induction. *n* = 3.	[41]	Figure 5Q
GSE113253	bulk RNA-seq	Human BM-MSCs: non-induced control, 9 d osteogenic induction, 9 d adipogenic induction. *n* = 3.	[29]	Figure 5R
GSE165771	bulk RNA-seq	HEK293 cells: mock control or *SP1* knockdown. *n* = 2.	[42]	Figure 8L

Table S2: Antibodies Used in This Study

Antibodies	Source	Catalog No.	Application
FITC-conjugated Anti-Mouse CD31	Invitrogen, Carlsbad, CA	11-0311-82	FCM
APC-conjugated Anti-Mouse CD34	Elabscience, Wuhan, China	E-AB-F1284E	FCM
APC-conjugated Anti-Mouse CD45	BioLegend, San Diego, CA	103111	FCM
APC-conjugated Anti-Mouse CD73	BioLegend	127209	FCM
APC-conjugated Anti-Mouse CD90	BioLegend	140311	FCM
APC-conjugated Anti-Mouse CD105	BioLegend	120413	FCM
GAPDH	Proteintech, Rosemont, IL	10494-1-AP	WB
PIEZO1	Proteintech	15939-1-AP	WB, IF
STC2	Proteintech	10314-1-AP	WB, IF
ALP	Affinity, Cincinnati, OH	DF6225	WB
RUNX2	Abcam, Cambridge, UK	ab236639	WB

**Antibodies**	**Source**	**Catalog No.**	**Application**
Osteocalcin	Abcam	ab93876	WB
PPARγ	Proteintech	16643-1-AP	WB
FABP4	Proteintech	12802-1-AP	WB
Histone H3	Proteintech	17168-1-AP	WB
SP1	Proteintech	21962-1-AP	WB
SP1	Sigma-Aldrich, St. Louis, MO	07-645	ChIP
Phospho-SP1 (Thr453)	Affinity	AF3121	WB
CaMKII-α	CST, Danvers, MA	#50049	WB
Phospho-CaMKII (Thr286)	CST	#12716	WB
CD73	Invitrogen	14-0731-82	IF
SP7 (Osterix)	Abcam	ab209484	IF
PPARγ	CST	#2435	IHC

Antibodies	Source	Catalog No.	Application
mCherry/tdTomato	HUABIO, Hangzhou, China	HA500049	IF
GFP	Abcam	ab13970	IF
HRP-conjugated Goat Anti-Mouse IgG (H+L)	Proteintech	SA00001-1	WB
HRP-conjugated Goat Anti-Rabbit IgG (H+L)	Proteintech	SA00001-2	WB
Donkey Anti-Rabbit IgG (H+L) Secondary Antibody, TRITC	Invitrogen	A16028	IF
Donkey Anti-Rabbit IgG (H+L) Secondary Antibody, Alexa Fluor 350	Invitrogen	A10039	IF
Donkey Anti-Rat IgG (H+L) Secondary Antibody, FITC	Invitrogen	A18746	IF
Donkey Anti-Chicken IgY (H+L) Secondary Antibody, Alexa Fluor 488	Invitrogen	A78948	IF

FCM: Flow cytometry, WB: Western blotting, IF: Immunofluorescence staining, IHC: Immunohistochemical staining, ChIP: Chromatin immunoprecipitation

Table S3: Primer Sequences Used for RT-qPCR

Primer	Sequences (5' to 3')
Gapdh-F	GTGTTCCTACCCCCAATGTGT
Gapdh-R	ATTGTCATACCAGGAAATGAGCTT
Piezo1-F	TCATCATCCTTAACCACATGGTG
Piezo1-R	TGAAGACGATAGCTGTCATCCA
Stc2-F	AGCAGGAAGTGTCCAGCAAT
Stc2-R	GGTTCACAAGGTCCACATAGG
Sp1-F	AGGGTCCGAGTCAGTCAGG
Sp1-R	CTCGCTGCCATTGGTACTGTT

F: Forward primer; R: Reverse primer

Table S4: siRNA Sequences Targeting Mouse *Sp1*

siRNA	Sequences (5' to 3')
si-Sp1-1-sense	GGAUGGUUCUGGUCAAAUA
si-Sp1-1-antisense	UAUUUGACCAGAACCAUCC
si-Sp1-2-sense	GGUGCAAACCAACAGAUCA
si-Sp1-2-antisense	UGAUCUGUUGGUUUGCACC
si-Sp1-3-sense	GGACAGACUCAGUAUGUGA
si-Sp1-3-antisense	UCACAUACUGAGUCUGUCC

Table S5: Primer Sequences Used for ChIP-PCR

**Primer**	**Sequences (5' to 3')**
Forward	CAGAGAAGCTGAACAGCTGCGGAA
Reverse	GATGAAACCTCCCAGGCGCATTAC

Table S6: Primer Sequences Used for Genotyping iCKO Mouse Lines

**Primer**	**Sequences (5' to 3')**
$\mathit{Rosa26}^{mTmG}$-P1	TAGAGCTTGCGGAACCCTTC
$\mathit{Rosa26}^{mTmG}$-P2	CTTTAAGCCTGCCCAGAAGA
$\mathit{Rosa26}^{mTmG}$-P3	AGGGAGCTGCAGTGGAGTAG
$\mathit{Piezo1}^{\mathrm{flox}}$-P1	GCCTAGATTCACCTGGCTTC
$\mathit{Piezo1}^{\mathrm{flox}}$-P2	GCTCTTAACCATTGAGCCATCT
$\mathit{Prrx1}^{CreERT2}$-P1	TCTCCTTTGTCCCTACAGCG
$\mathit{Prrx1}^{CreERT2}$-P2	CTCCGCCATGGTCCCAATA
$\mathit{Prrx1}^{CreERT2}$-P3	ACAGCGCCATGGCTACTTAT
$\mathit{Prrx1}^{CreERT2}$-P4	ATTCAACTTGCACCATGCCG
